# The crucial role of vascular tissues and COE2-mediated retrograde signaling in mitigating cadmium stress in *Arabidopsis* *thaliana*

**DOI:** 10.1007/s44154-024-00196-4

**Published:** 2025-02-06

**Authors:** Zhixin Liu, Yumeng Liu, Yaping Zhou, Hao Liu, Aizhi Qin, Luyao Kong, Lulu Yan, Chunyang Li, Peibo Gao, Qianli Zhao, Xiao Song, Mengmeng Zhou, Mengfan Li, Yajie Xie, Enzhi Guo, Meng Qin, Xuwu Sun

**Affiliations:** National Key Laboratory of Cotton Bio-Breeding and Integrated Utilization, State Key Laboratory of Crop Stress Adaptation and Improvement, School of Life Sciences, Henan University, Kaifeng, 475001 China

**Keywords:** Arabidopsis, Cadmium stress, Vascular tissue, Chloroplast, COE2

## Abstract

Cadmium is a heavy metal that significantly harms plant growth and development by impairing enzyme activity and photosynthesis, leading to chloroplast damage. In this study, we discovered that genes specifically expressed in vascular tissues regulate the plant’s response to heavy metals by scRNA-seq. The overexpression of vascular-specific genes such as *AHP1*, *DOF2.4*, *NHL1*, *CDF4*, and *ATARFB1A* increased tolerance to cadmium stress by maining the chloroplast functions. In addition, we found that two chloroplast proteins PSBO1 and J3 are involved in regulating the function of chloroplasts, starch metabolism, and leaf vein development in response to cadmium stress. Further analysis revealed that COE2-mediated retrograde signaling protects chloroplast structure and function by regulating the expression of *J3* and *PsbO1*, and managing the quality control of damaged chloroplasts via *FC1* and *PUB4*. These findings provide insights into the mechanisms plants use to counteract the detrimental effects of cadmium stress.

**Supplementary Information:**

The online version contains supplementary material available at 10.1007/s44154-024-00196-4.

## Introduction

Heavy metals, characterized by their high density (Jaiswal et al. 2018), are increasingly recognized as significant environmental pollutants and global public health threats (Pandiyan et al. 2021; Pozgajova et al. 2022; Rakib et al. 2022). These metals are pervasive and pose serious risks to organisms, microorganisms, and the environment (Li et al. 2017; Wang et al. 2021), affecting cellular components such as lysosomes, endoplasmic reticulum, cell membranes, mitochondria, nuclei, and enzymes involved in metabolism, detoxification, and damage repair (Wang and Shi 2001; El-Okkiah et al. 2022). In plants, heavy metals can cause dwarfism, slow growth, leaf yellowing, and death (Saud et al. 2022).

Cadmium, for example, disrupts plant metabolism, leading to stunted growth, slow development, reduced yield, imbalanced nutrient metabolism, and death (Nagajyoti et al. 2010). Plants absorb cadmium through their roots via apoplastic and symplastic pathways, then transfer it to shoots through the xylem, and finally to edible parts through the phloem (Zhang et al. 2020; Ur Rahman et al. 2021). Vascular tissues play a crucial role in this process, as they provide structural support and transport essential substances for growth and defense (Dettmer et al. 2009). Cadmium accumulation disrupts physiological and biochemical processes, altering the morphology of nutritional and reproductive (Rehman et al. 2018). In particular, cadmium inhibits root and shoot growth, photosynthetic activity, stomatal conductance, and overall plant biomass (Wang et al. 2022), with a more pronounced effect on male reproductive parts, thereby impacting fruit growth (Shirkhani et al. 2020).

Excessive accumulation of cadmium in plant organs inhibits growth (Hladun et al. 2015). For example, in radish stems, it reduces the fresh weight of shoots and adversely affects anatomical structures by expanding thin-walled cells in the cortex, endodermis, and pericycle region, and damaging root cells (Chaca et al. 2014). Increased cadmium concentrations have been found to decrease shoot growth, leaf area, and hydraulic conductivity in European blueberries (Hatamian et al. 2020), as well as reduce stem and root growth in flowering plants (Tauqeer et al. 2016). These effects illustrate how cadmium impacts root elongation, lateral root branching, and root tip growth.

Photosynthesis is also affected by cadmium stress. Cadmium interferes with chlorophyll synthesis by replacing magnesium ions, altering chlorophyll concentration, and restricting the photochemical activity of Photosystem II (PSII) (Parmar et al. 2013; Dobrikova et al. 2017). It impacts chloroplast ultrastructure, reducing stomatal conductance, leaf transpiration, and gas exchange (Najeeb et al. 2011; Andresen et al. 2020). Cadmium causes stomatal closure and reduces CO_2_ conductance, impairing photosynthetic function (Nikolic et al. 2017). Furthermore, cadmium inhibits photosynthesis by reducing gene transcription, deactivating enzymes involved in CO_2_ fixation, inducing lipid peroxidation, protein hydrolysis, and disrupting nitrogen and sulfur metabolism (Gallego et al. 2012).

Plants have developed mechanisms to mitigate cadmium stress. In Arabidopsis, enhanced expression of the *OsHarbinger Transposase Derived 1–1* (*OsHARBI1-1*) increases cadmium tolerance, leading to increased cadmium accumulation in roots while slightly reducing it in aboveground parts (Jiang et al. 2023). In poplar, the Abscisic Acid Responsive Element Binding Protein (PtrAREB3) enhances cadmium tolerance by reducing oxidative stress, minimizing chlorophyll degradation, increasing proline synthesis, and activating antioxidant systems (Shi et al. 2023). In wheat, the transcription of Copper Transporter 3D (TaCOPT3D) under cadmium stress accumulates in roots, reducing reactive oxygen species and increasing antioxidant enzyme activities (Liu et al. 2022a). Additionally, COPPER TRANSPORTER 3 (COPT3) and ZINC TRANSPORTER 1 (ZnT1) have been identified as major cadmium transport proteins in wheat (Zheng et al. 2023).

Recent research has shown that heavy metal-associated isoprenylated plant proteins (HIPPs) play a crucial role in regulating cadmium in plants (de Abreu-Neto et al. 2013). HIPPs are metal-binding proteins characterized by heavy metal-associated (HMA) domains and isoprenylation motifs (Barth et al. 2009). To date, 45, 59, and 74 HIPP genes have been identified in Arabidopsis, rice, and poplar, respectively. These genes fall into five distinct clusters. All HIPP proteins share conserved structures, including the HMA domain and C-terminal isoprenylated CaaX motif, with some also featuring glycine-rich repeats and proline-rich motifs (de Abreu-Neto et al. 2013).

Adverse environmental conditions affect the expression of *HIPP* genes. For example, *OsHIPP41* shows high expression in rice under drought and cold conditions, while *OsHIPP28*, *OsHIPP41*, and *OsHIPP21* significantly increase in seedlings exposed to cadmium (de Abreu-Neto et al. 2013). In Arabidopsis, the expression of *HIPP6* is induced by Cd, Hg, Fe, and Cu, and the expression of *HIPP20*, *HIPP22*, *HIPP26*, and *HIPP27* in yeast enhances cadmium resistance in cadmium-sensitive yeast strains. The *hipp20/21/22* triple mutant exhibits increased sensitivity to cadmium stress (Suzuki et al. 2002). AtHIPP44 in Arabidopsis interacts with the transcription factor MYB49, significantly upregulating its expression to reduce cadmium accumulation (Zhang et al. 2019). In rice, the *osHIPP42* mutant shows weaker growth under Mn, Cd, and Cu stress, with induced expression of *OsHIPP16*, *OsHIPP28*, *OsHIPP34*, *OsATX1*, and *OsHIPP60* in roots and shoots (Khan et al. 2019). In tea, overexpression of *CsHIPP22*, *CsHIPP24*, and *CsHIPP36* in the yeast mutant *ycf1* enhances cadmium resistance (Wei et al. 2023). These findings suggest that *HIPP* genes are vital for cadmium detoxification in plants (Tehseen et al. 2010).

Identifying genes that respond to cadmium stress in specific tissues and cell types is crucial for understanding the precise regulation of these responses. The application of single-cell RNA sequencing (scRNA-seq) in plant research has enabled the identification of genes expressed in particular tissues and cells. For instance, scRNA-seq analysis of 5-day-old Arabidopsis cotyledons revealed the transcriptional network regulating the development of meristematic mother cells into guard mother cells during stomatal development (Liu et al. 2020). Further scRNA-seq analysis identified roles for the *CYCLING DOF FACTOR 5* (*CDF5*) and *REPRESSOR OF GA* (*RGA*) genes in the early development and function of cotyledon veins (Liu et al. 2022c). Additionally, scRNA-seq identified the expression patterns of *PHYTOCHROME INTERACTING FACTOR 1* (*PIF1*), *PIF3*, *PIF4*, and *PIF5* genes in Arabidopsis epidermal and guard cells, suggesting their involvement in regulating cell development under stress conditions (Liu et al. 2022b).

In previous study we identified the *HIPP36* gene as being specifically expressed in vascular tissues (Liu et al. 2022c). Here, we investigated the roles of genes that expressed in vascular tissues and found that cadmium stress inhibit seedlings growth and development by damaging vein development, reduce photosynthetic function and starch metabolism. The study revealed that overexpressing vascular-specific genes enhances seedling resistance to cadmium stress, maintains chloroplast photosynthetic function, and supports root development. Further analysis showed that chloroplast genes *J3* and *PSBO1* play a role in regulating seedling resistance to cadmium stress. Additionally, COE2-mediated plastid retrograde signaling regulates the quality control of thylakoid membrane by modulating the expression of *FC1* and *PUB4* under cadmium stress. This results uncovers potential mechanisms by which plants coordinate vascular tissue development and chloroplast function to counteract cadmium stress, highlighting the intricate balance between these systems in enhancing plant resilience.

## Results

### Single-cell transcriptome sequencing identified genes in vascular tissue cells that respond specifically to cadmium stress

**Fig. 1:**
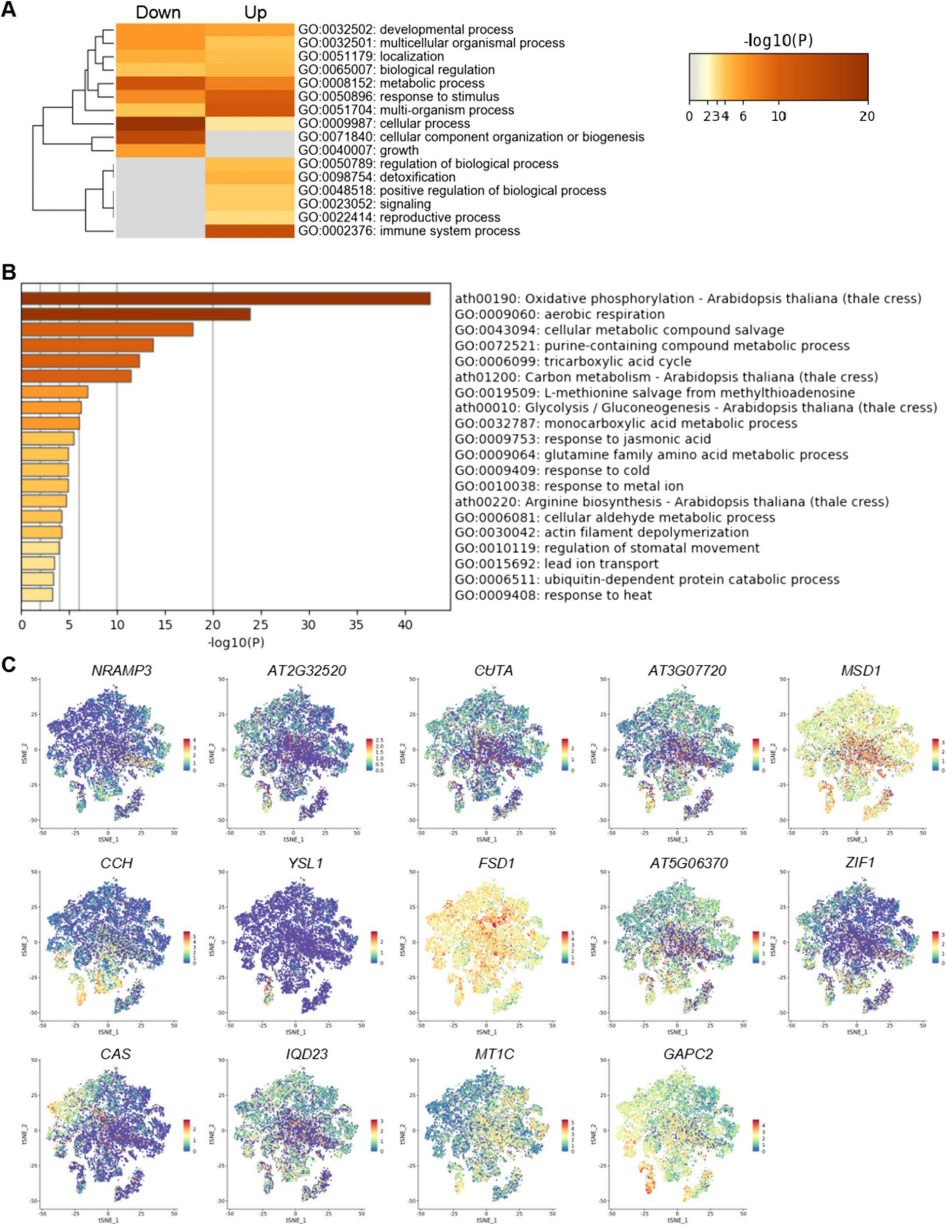
Identification of cadmium stress responsive genes and visualization analysis of cell types. **A** Gene Ontology (GO) enrichment analysis of differentially expressed genes under cadmium stress treatment. “Down” represents genes with expression levels downregulated under cadmium stress conditions, while “Up” represents genes with expression levels upregulated under cadmium stress conditions. **B** GO enrichment analysis of genes specifically expressed in companion cells from single-cell transcriptome data. **C** Visualization of cell expression of 14 genes specifically induced by cadmium stress

In our previous study on *Arabidopsis* cotyledons, we found that *HEAVY METAL ASSOCIATED ISOPRENYLATED PLANT PROTEIN 36* (*HIPP36*), a gene known for its role in heavy metal homeostasis and detoxification(Zhang et al. 2020), is highly expressed in companion cell (Liu et al. 2022c). This suggests that genes expressed in vascular tissue cells, such as those in the cortex, may regulate plant responses to heavy metal stress. To screen for genes induced by heavy metal stress and their cell-specific expression, we analyzed published RNA-seq data induced by Cd^2+^ stress (Fischer et al. 2017). We identified 872 down-regulated and 745 up-regulated genes under cadmium stress (Table S1). Gene Ontology (GO) analysis of these genes revealed that up-regulated genes are enriched in terms such as “response to stimulus,” “detoxification,” “positive regulation of biological process,” “reproductive process,” and “immune system process” (Fig. 1 A and Table S2). In contrast, down-regulated genes are enriched in “metabolic process,” “cellular process,” and “cellular component organization or biogenesis” (Fig. 1 A).

To investigate the cell-specific expression of genes induced by cadmium treatment, we selected representative genes for expression pattern analysis in the single-cell data. Genes such as *NATURAL RESISTANCE-ASSOCIATED MACROPHAGE PROTEIN 3* (*NRAMP3*), *AT2G32520*, *CUTA DIVALENT CATION TOLERANCE HOMOLOG* (*CUTA*), *AT3G07720*, *MANGANESE SUPEROXIDE DISMUTASE 1* (*MSD1*), *COPPER CHAPERONE* (*CCH*), *YELLOW STRIPE LIKE 1* (*YSL1*), *FE SUPEROXIDE DISMUTASE 1* (*FSD1*), *AT5G06370*, *ZINC INDUCED FACILITATOR 1* (*ZIF1*), *CALCIUM SENSING RECEPTOR* (*CAS*), *IQ-DOMAIN 23* (*IQD23*), *METALLOTHIONEIN 1C* (*MT1C*), and *GLYCERALDEHYDE-3-PHOSPHATE DEHYDROGENASE C2* (*GAPC2*) were significantly expressed in companion cell (Fig. 1 C and Table S3). GO analysis of the differentially expressed genes (DEGs) in companion cell showed enrichment in terms such as “response to metal ion,” “response to cold,” and “cellular aldehyde metabolic process” (Fig. 1B and Table S3). The “response to metal ion” term in cell cluster 8 shares similarities with the cadmium-induced gene GO term “detoxification,” indicating significant expression of these cadmium-induced genes in companion cell. This suggests that vascular tissues play a role in the response to cadmium stress.

To further confirm the involvement of vascular tissues in the response to cadmium stress, we analyzed the relative expression levels of several cadmium-affected genes in vascular tissues under different conditions. As shown in Fig.S1, compared to normal conditions, the expression of genes *NRAMP3*, *AT2G32520*, *GAPC2*, *IQD23*, *MT1C*, and *MSD1* was suppressed under cadmium stress, while the expression of genes *YSL1* and *ZIF1* increased. These results indicate that cadmium stress affects gene expression in vascular tissues.

### Effects of CdCl_2_ on Arabidopsis leaf vein development

To investigate how Arabidopsis responds to cadmium stress, we examined the impact of CdCl_2_ on leaf vein development. Treatment with 70 μM CdCl_2_ altered leaf vein formation compared to the control, indicating that CdCl_2_ affects vascular development in Arabidopsis (Fig.S2).

We then analyzed the expression of specific vascular marker genes to identify those involved in the cadmium-induced inhibition of vascular development. The genes *HISTIDINE-CONTAINING PHOSPHOTRANSMITTER 1* (*AHP1*), *DNA BINDING WITH ONE FINGER 2.4* (*DOF2.4*), *NHL1*, *CYCLING DOF FACTOR 4* (*CDF4*), and *ADP-RIBOSYLATION FACTOR B1A* (*ATARFB1A*) were significantly expressed in specific cell clusters (Phloem parenchyma, Xylem parenchyma, and companion cell) under CdCl_2_ treatment (Fig.S3 A). These genes were markedly upregulated compared to normal conditions (Fig.S3 B).

**Fig. 2:**
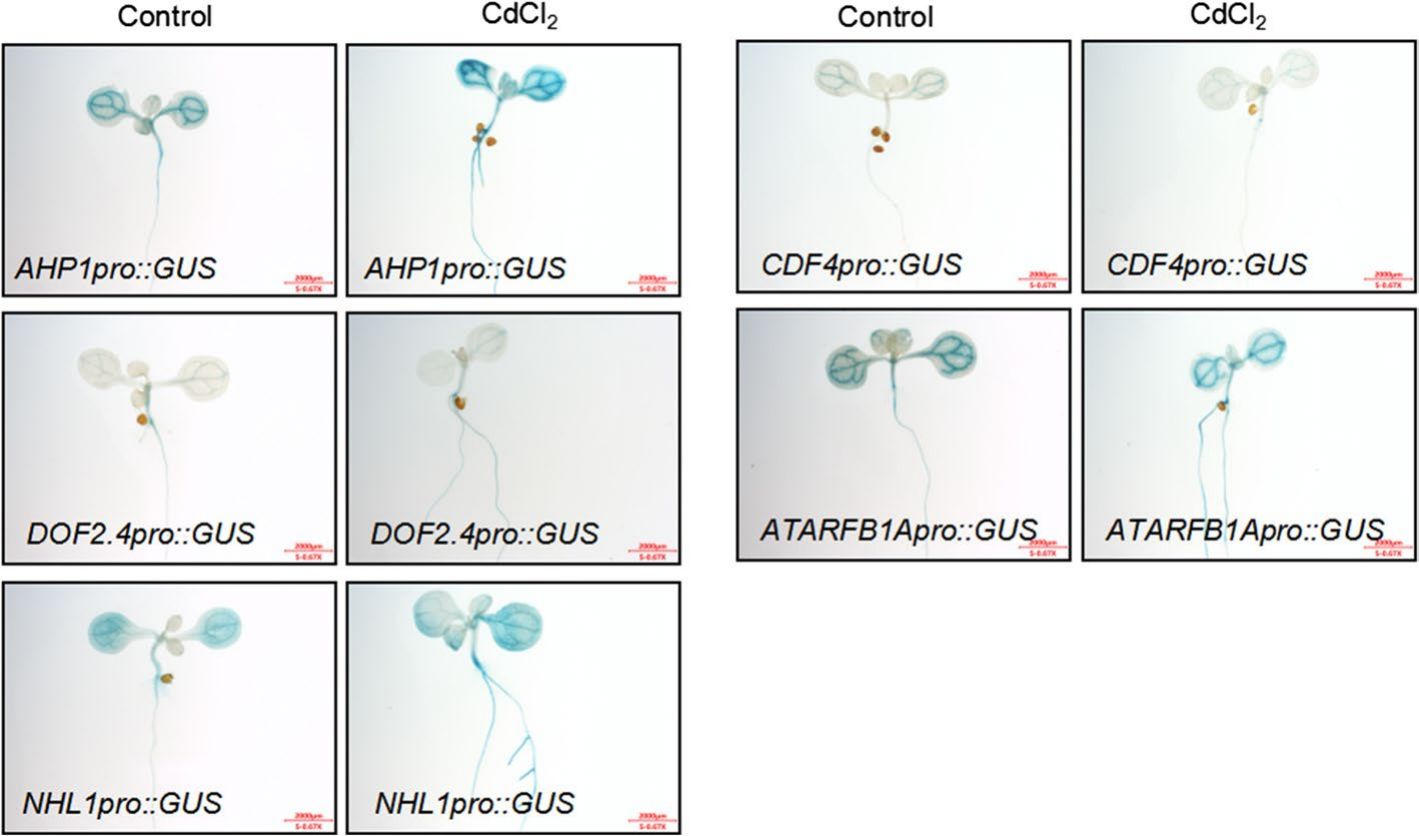
GUS staining analysis of the expression patterns of vascular tissue-specific expressed genes. To analyze the effect of CdCl_2_ on the expression patterns of vascular specific expression genes, we generated transgenic plants with GUS reporter genes driven by their own promoters, including *AHP1pro::GUS*, *DOF2.4pro::GUS*, *NHL1pro::GUS*, *CDF4pro::GUS*, and *ATARFB1Apro::GUS*. The expression patterns of these genes under control and CdCl_2_ conditions were analyzed by GUS staining. Scale bar = 2 m m

To further understand how CdCl_2_ affects gene expression, we created transgenic plants with GUS reporter genes under the control of the promoters for these marker genes. GUS staining revealed increased activity of *AHP1pro::GUS* and *NHL1pro::GUS* in leaf veins and roots following CdCl_2_ treatment. Conversely, GUS staining for *DOF2.4pro::GUS*, *CDF4pro::GUS*, and *ATARFB1Apro::GUS* diminished in leaves but increased in roots under the same conditions (Fig. 2). This suggests that cadmium stress alters the expression patterns of these vascular marker genes.

### Vascular-specific expressed genes involved in response to CdCl_2_ treatment

**Fig. 3:**
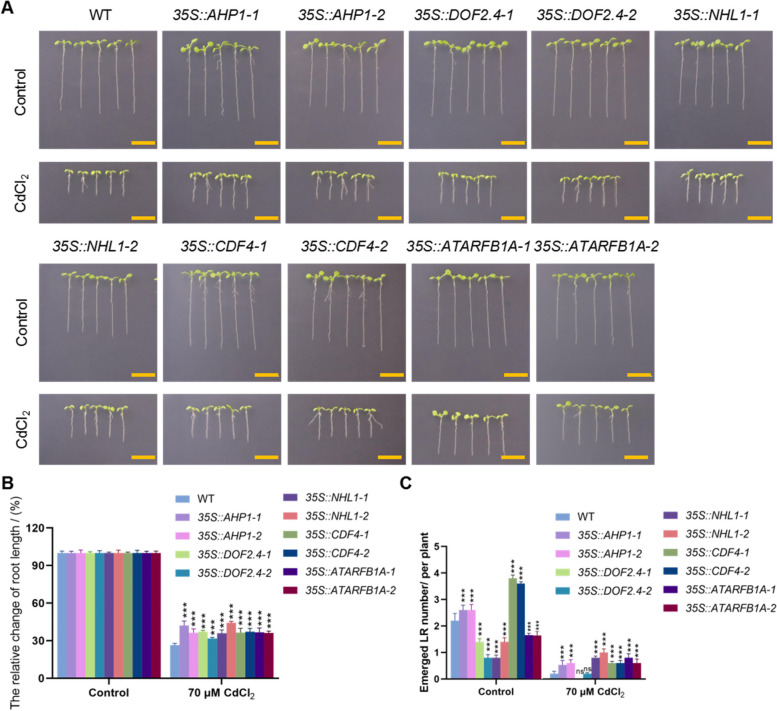
Growth and developmental phenotypes of seedlings overexpressing genes specific to microtubule tissue under normal and cadmium stress conditions. **A** The growth and developmental phenotypes of WT, *35S::AHP1*, *35S::DOF2.4*, *35S::NHL1*, *35S::CDF4*, and *35S::ATARFB1A* overexpressing seedlings after 7 days of growth on normal and 1/2MS medium supplemented with 70 μM CdCl_2_. Scale bar = 1 cm. **B** The relative percentage of changes in seedling root length; The error line represents the standard deviation, and three biological replicates (*n* = 5) were performed to determine the significant difference between wild-type and overexpressing plants through one-way ANOVA, *** *p* < 0.001. **C** Statistical analysis of lateral root numbers in seedlings; Perform 3 biological replicates (*n* = 5) and determine the significant differences between WT and overexpressing plants through one-way ANOVA, *** *p* < 0.001

Previous analyses have shown that CdCl_2_ promotes the expression of vascular marker genes. To assess the roles of these CdCl_2_-induced genes in regulating cadmium stress, we generated overexpression plants for *AHP1*, *DOF2.4*, *NHL1*, *CDF4*, and *ATARFB1A* under the 35S promoter (*35S::AHP1*, *35S::DOF2.4*, *35S::NHL1*, *35S::CDF4*, and *35S::ATARFB1A*) (Fig.S4). Compared to the control, root length significantly decreased and lateral roots were notably reduced in both wild type (WT) and overexpression seedlings under CdCl_2_ conditions (Fig. 3). The percentage change in root length was significantly greater in the overexpression plants than in WT under CdCl_2_ conditions (Fig. 3 A and B). For lateral roots, compared to WT under control conditions, *35S::AHP1* and *35S::CDF4* showed significantly more lateral roots, while *35S::DOF2.4, 35S::ATARF1A* and *35S::NHL1* exhibited significantly fewer (Fig. 3 A and C). Under CdCl_2_ treatment, lateral root development in all overexpression lines displayed better than WT, except *35S::DOF2.4* (Fig. 3 A and C).

### CdCl_2_ Impact on leaf vein development

**Fig. 4:**
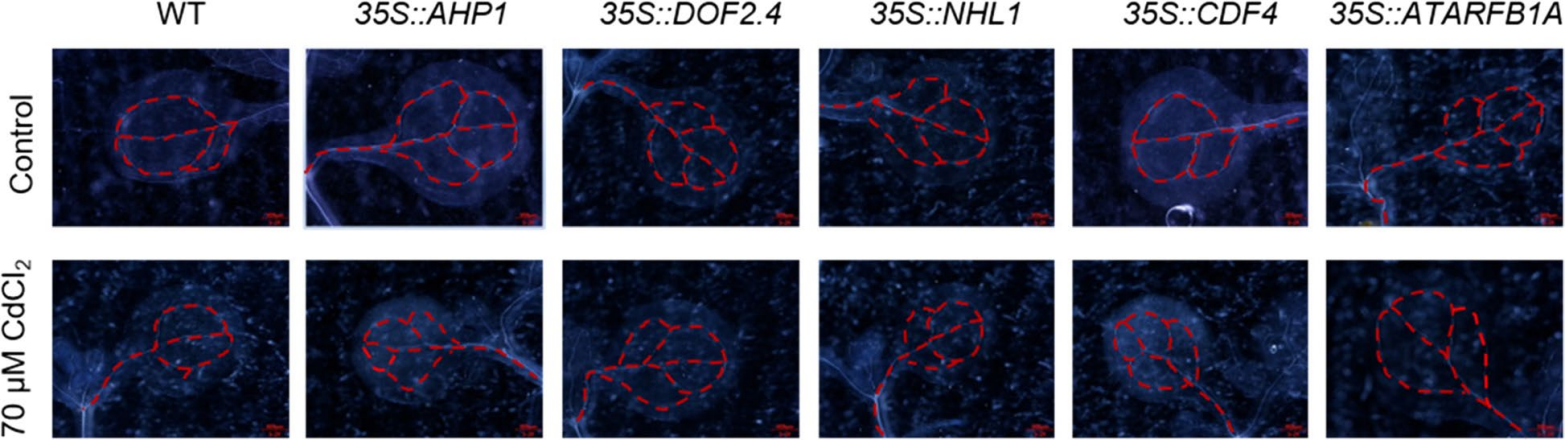
Analysis of the developmental status of leaf veins in the cotyledons of Arabidopsis seedlings of WT and transgenic lines with overexpression of vascular bundle specific expression genes under normal and cadmium stress conditions. Overexpressed Arabidopsis seedlings (*35S::AHP1*, *35S::DOF2.4*, *35S::NHL1*, *35S:: CDF4*, and *35S::ATARFB1A*) and WT were grown on 1/2 MS medium plate and 1/2MS medium plate plus CdCl_2_, and then fixed with chloral hydrate to observe the development of cotyledon veins under a phase contrast microscope. Cotyledon veins were marked with red dashed lines. Scale bar = 0.5 mm

CdCl_2_ significantly affected leaf development. Leaf veins are a major site of vascular bundle development, so we analyzed the developmental status of leaf veins in WT and overexpression plants after 7 days of growth on control and CdCl_2_-supplemented media. As shown in Fig. 4, after CdCl_2_ treatment, WT showed slowed leaf vein development, forming only one large vascular loop, whereas under control conditions, a second layer of vascular loops developed. In contrast, there were no significant changes in leaf vein development in the overexpression lines under control and CdCl_2_ conditions.

### Effect of CdCl_2_ on Chlorophyll fluorescence in Arabidopsis

**Fig. 5:**
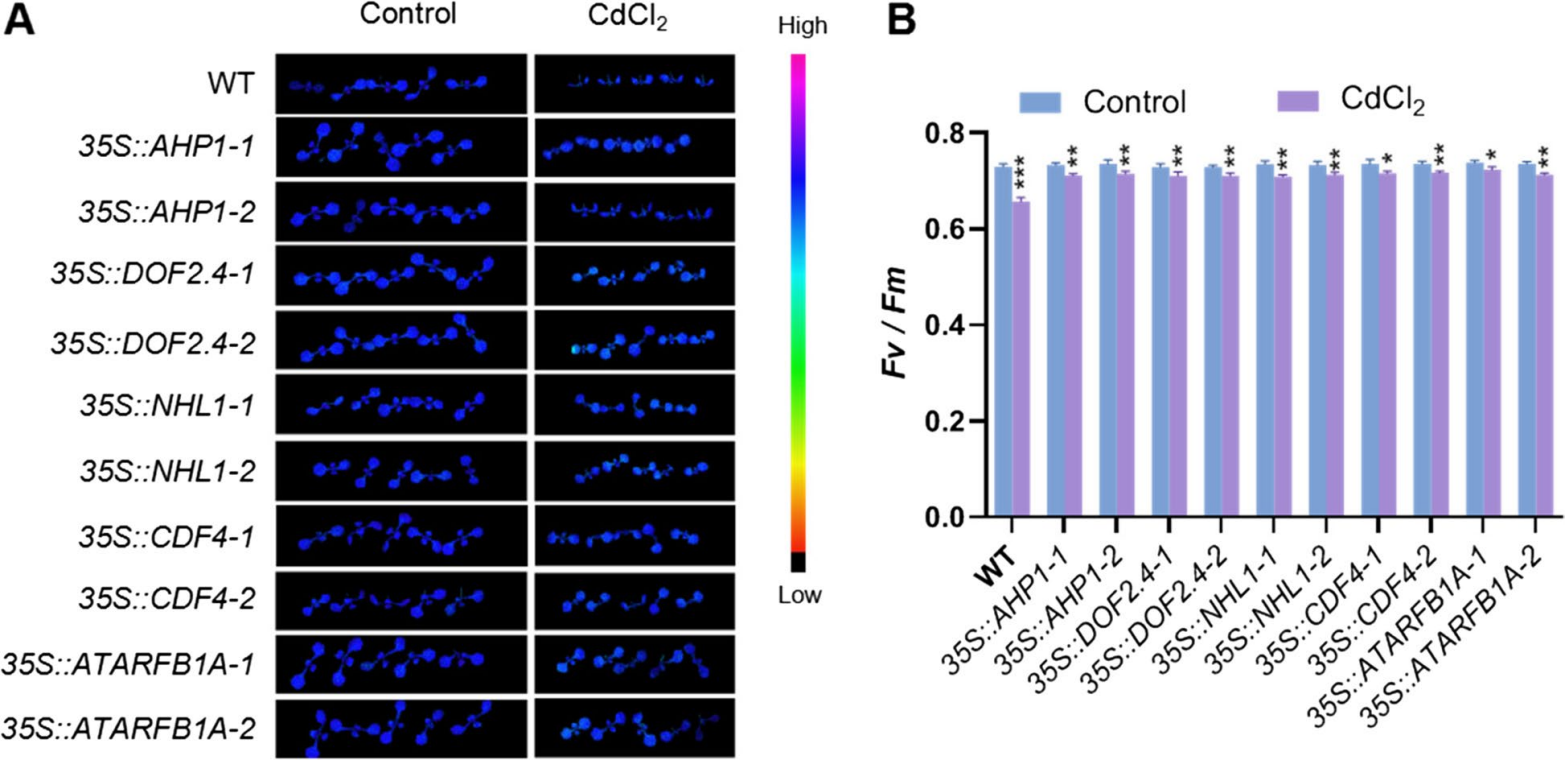
Chlorophyll fluorescence measurement and statistical analysis of WT and *35S::AHP1*, *35S::DOF2.4*, *35S::NHL1*, *35S::CDF4*, and *35S:: ARARFB1A* seedlings. **A** In order to observe the effect of CdCl_2_ on plant photosynthesis, a chlorophyll fluorescence meter was used to measure the chlorophyll fluorescence of WT and *35S:: AHP1*, *35S::DOF2.4*, *35S::NHL1*, *35S::CDF4*, and *35S::ARARFB1A* seedlings grown on 1/2 MS medium plate (control) and 1/2 MS medium plate plus CdCl_2_ for 7 days. The image results of their Fv/Fm values are shown in the figure. **B** Statistical analysis was conducted on the Fv/Fm values in (A), and the error line represents the standard deviation. The Fv/Fm values under normal conditions were used as controls. Student’s t-test was used to analyze the significant differences between cadmium stress conditions and normal conditions (*n* = 5), with ** *p* < 0.001, ** *p* < 0.01, ** *p* < 0.05

Studies have indicated that cadmium stress affects plant photosynthesis(Gao et al. 2019). To investigate the impact of CdCl_2_ on photosynthesis in WT and overexpression lines, we conducted chlorophyll fluorescence analysis on seedlings grown for 7 days under control and CdCl_2_-treated conditions. The results showed that under control conditions, there were no significant differences in Fv/Fm between the overexpression lines and WT (Fig. 5). However, after CdCl_2_ treatment, Fv/Fm decreased in all seedlings, with the overexpression lines maintaining significantly higher Fv/Fm compared to WT (Fig. 5). These results suggest that overexpression of vascular-specific genes may enhance resistance to CdCl_2_ stress by improving photosynthetic function, potentially through enhanced chloroplast function and increased metabolic activity and stress tolerance.

### Chloroplast genes and CdCl_2_ stress response

To explore the impact of chloroplast photosynthesis on seedling resistance to CdCl_2_ stress, we selected the *DNAJ HOMOLOGUE 3* (*J3*) and *PS II OXYGEN-EVOLVING COMPLEX 1* (*PsbO1*) genes, which are expressed in vascular tissues and involved in chloroplast function(Takahashi et al. 2017; Zhou et al. 2018). Single-cell data analysis showed significant expression of *J3* and *PsbO1* in Bundle sheath, companion cell, Xylem parenchyma, and Phloem parenchyma cells (Fig.S5 A). Under CdCl_2_ stress, expression of *PSBO1* and *J3* increased (Fig.S5 B).

**Fig. 6:**
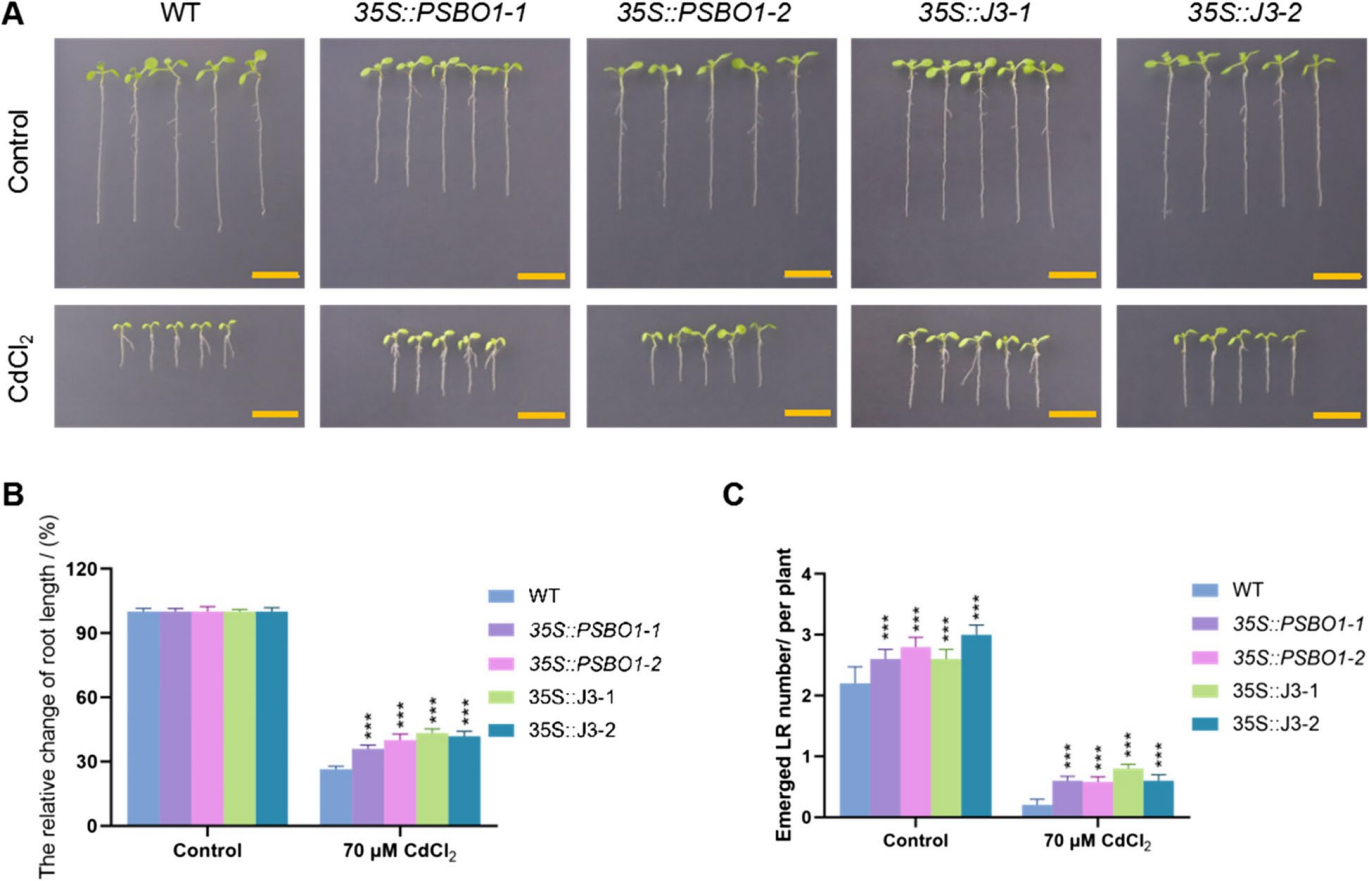
Growth and developmental phenotypes of WT, *35S::PSBO1-1*, *35S::PSBO1-2*, *35S::J3-1*, and *35S::J3-2* seedlings under normal and cadmium stress conditions. **A** The growth and developmental phenotypes of WT, *35S::PSBO1-1*,*35S::PSBO1-2*, *35S::J3-1*, and *35S::J3-2* seedlings after 7 days of growth on normal and 1/2MS medium supplemented with 70 μM CdCl_2_. Scale bar = 1 cm. **B** The relative percentage of changes in seedling root length; The error line represents the standard deviation, and three biological replicates were performed to determine the significant difference between wild-type and overexpressing plants through one-way ANOVA, *** *p* < 0.001. **C** Statistical analysis of lateral root numbers in seedlings; 3 biological replicates (*n* = 5) and determine the significant differences between WT and overexpressing plants through one-way ANOVA, *** *p* < 0.001

We generated overexpression plants for these genes (*35S::PSBO1-1*, *35S::PSBO1-2*, *35S::J3-1*, and *35S::J3-2*) to study their roles in regulating growth and development under CdCl_2_ stress (Fig.S6). Compared to WT, root length and lateral root numbers significantly decreased in all seedlings under CdCl_2_ treatment (Fig. 6). However, the primary root length was longer in the overexpression lines than in WT after CdCl_2_ treatment (Fig. 6 A and B). Under both control and CdCl_2_ treatment conditions, lateral roots were more abundant in the overexpression lines compared to WT (Fig. 6 A and C), indicating reduced sensitivity to CdCl_2_ stress.

Chlorophyll fluorescence analysis revealed that under control conditions, there were no significant differences in Fv/Fm between the overexpression lines and WT. After CdCl_2_ treatment, Fv/Fm was significantly higher in the overexpression lines compared to WT (Fig.S7 A and B).

### CdCl_2_ affects chloroplast starch synthesis and metabolism

**Fig. 7:**
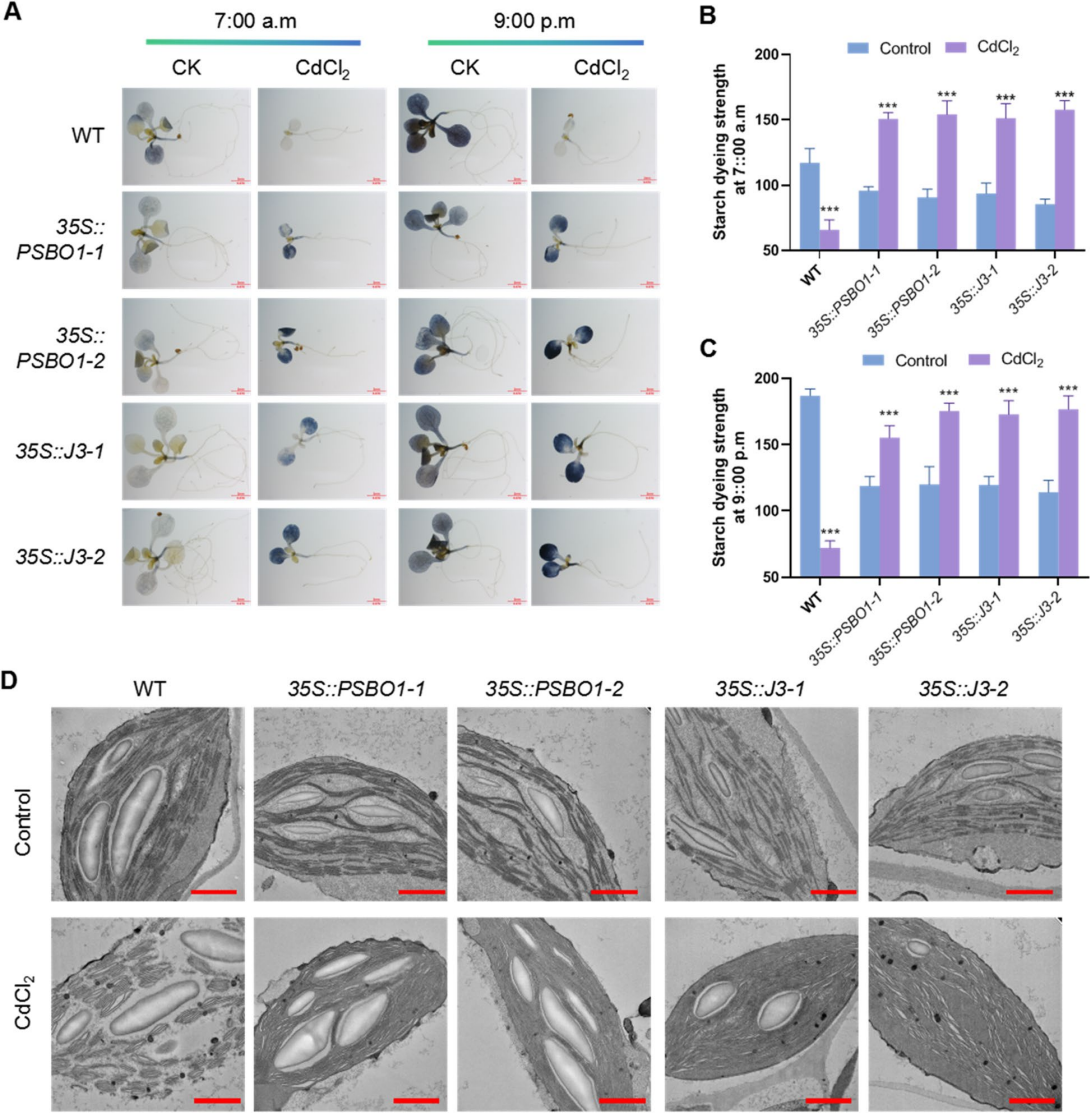
Analysis of the effects of CdCl_2_ treatment on the synthesis and metabolism of starch in WT, *35S:: PSBO1*, and *35S:: J3* seedlings. **A** Analyze the effects of CdCl_2_ on starch synthesis and metabolism in WT, *35S:: PSBO1-1*, *35S:: PSBO1-2*, *35S:: J3-1*, and *35S:: J3-2* seedlings using I_2_/KI staining method. All seedlings were grown on 1/2 MS medium plate (control) and 1/2MS medium plate plus CdCl_2_ for 7 days, and the materials were taken at 7 am and 9 pm. After decolorization with 70% alcohol, starch staining was detected using a stereomicroscope at a scale of 2 mm. **B** Statistics on starch staining intensity of WT, *35S::PSBO1-1*, *35S::PSBO1-2*, *35S::J3-1*, and *35S::J3-2* seedlings at 7am; The error line represents the standard deviation, and the significance of the difference under CdCl_2_ conditions compared to normal conditions was analyzed using Student’s t-test (*n* = 3), *** *p* < 0.001. **C** At 9 pm, the starch staining intensity of WT, *35S::PSBO1-1*, *35S::PSBO1-2*, *35S::J3-1*, and *35S::J3-2* seedlings was statistically analyzed. The error line represents the standard deviation. Student’s t-test was used to analyze the significance of the difference between CdCl_2_ conditions and normal conditions (*n* = 3), *** *p* < 0.001. **D** Using transmission electron microscopy to detect and analyze the chloroplasts of WT, *35S:: J3-1*, *35S:: J3-2*, *35S:: PSBO1-1*, and *35S:: PSBO1-2* seedlings under control and CdCl_2_ conditions. Scale bar = 1 μ m

To analyze the impact of CdCl_2_ on photosynthetic function in Arabidopsis seedlings, we measured starch content in WT and overexpression lines (*35S::PSBO1-1*, *35S::PSBO1-2*, *35S::J3-1*, and *35S::J3-2*). Under CdCl_2_ conditions, WT showed significantly reduced starch staining levels both before lights on (7:00 a.m.) and after lights off (9:00 p.m.), indicating an effect on starch metabolism and synthesis (Fig. 7). In contrast, the overexpression lines exhibited significantly increased starch staining levels after CdCl_2_ treatment. Compared to WT, starch staining levels were lower in the overexpression lines under control conditions but significantly higher after CdCl_2_ treatment (Fig. 7). Additionally, under control conditions, both WT and transgenic lines showed a significant decrease in starch content after overnight consumption. After CdCl_2_ treatment, WT did not show significant starch synthesis or metabolism during the day and night, whereas starch levels in the overexpression lines remained high before and after lights off (Fig. 7). These results suggest that under CdCl_2_ conditions, although starch can be synthesized, its degradation activity is reduced. This reduced degradation activity may contribute to the slowed growth rate of seedlings under cadmium stress.

### CdCl_2_ stress disrupts chloroplast structure

Under CdCl_2_ conditions, reduced chlorophyll fluorescence activity and weakened starch degradation activity suggest possible impairment of chloroplast function. To explore the potential reasons for CdCl_2_-induced effects on chloroplast function, we observed chloroplast ultrastructure in WT, *35S::PSBO1-1*, *35S::PSBO1-2*, *35S::J3-1*, and *35S::J3-2* grown under control and CdCl_2_ conditions using transmission electron microscopy (TEM). As shown in Fig. 7, compared to control, CdCl_2_ treatment led to structural damage of thylakoid membranes in WT chloroplasts, with observed loosening of grana stacks. In contrast to WT, chloroplast thylakoid membrane structures and grana stacks in *35S::PSBO1-1*, *35S::PSBO1-2*, *35S::J3-1*, and *35S::J3-2* seedlings showed less than WT under control conditions (Fig. 7 D). Under CdCl_2_ conditions, while the thylakoid membrane structures in *35S::PSBO1-1*, *35S::PSBO1-2*, *35S::J3-1*, and *35S::J3-2* seedlings were relatively intact, there was a significant reduction in grana stacking compared to WT (Fig. 7). These results indicate that CdCl_2_ stress disrupts chloroplast structure, thereby affecting photosynthetic function. Despite minimal damage to thylakoid membrane structures in *35S::PSBO1-1*, *35S::PSBO1-2*, *35S::J3-1*, and *35S::J3-2* seedlings under CdCl_2_ conditions, the significant reduction in grana stacking could affect photosynthetic function and starch metabolism activity under cadmium stress.

### Effects of CdCl_2_ on Chloroplast gene expression and vein development in Arabidopsis

**Fig. 8:**
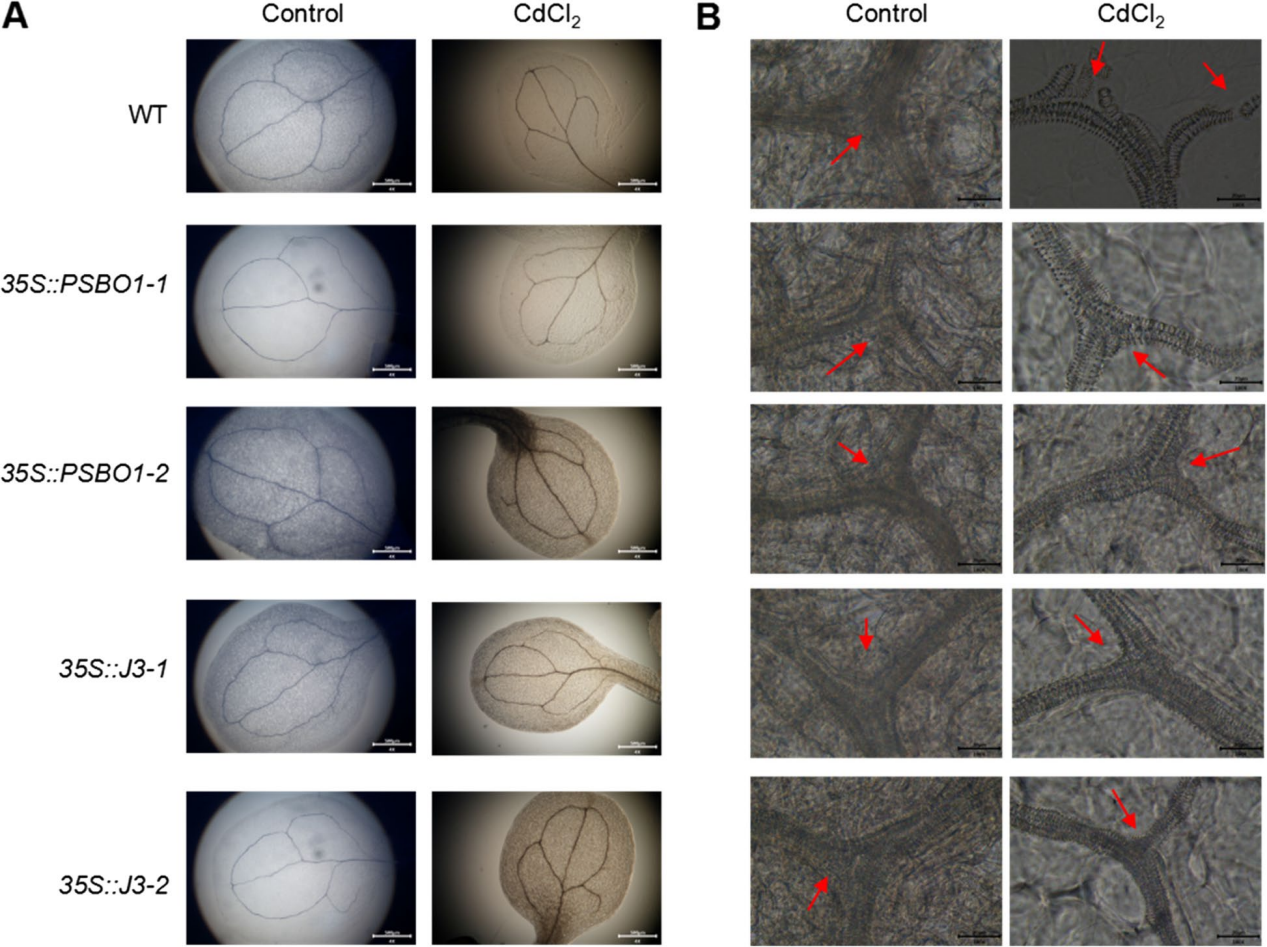
Analysis of the effects of CdCl_2_ treatment on the development of leaf vein tissues in WT, *35S:: PSBO1*, and *35S:: J3* seedlings. **A** The cotyledons of WT, *35S::J3-1*, *35S::J3-2*, *35S::PSBO1-1*, and *35S:: PSBO1-2* seedlings grown for 7 days under control and CdCl_2_ conditions were transparent with chloral hydrate, and their vein development status were detected with a phase contrast microscope. Scale bar = 500 μm. **B** Observe the local part of the leaf vein tissue in (A) with the red arrow indicating the leaf vein tissue cells. Scale bar = 20 μm

To investigate the impact of CdCl_2_ on chloroplast gene expression and vein development in Arabidopsis, we compared vein structures in WT plants and transgenic lines (*35S::PSBO1-1*, *35S::PSBO1-2*, *35S::J3-1*, and *35S::J3-2*) grown under both control and CdCl_2_ conditions. Our observations revealed that, under CdCl_2_ stress, the WT plants exhibited significant vein damage, including fractures at vein bifurcations. In contrast, the transgenic lines showed no notable changes in vein development (Fig. 8 A).

Additionally, detailed observations of vessel structures revealed that WT plants under CdCl_2_ stress experienced significant loosening of vessel connections and bundle breakage. The transgenic lines, however, did not show similar damage (Fig. 8 B). These findings indicate that CdCl_2_ disrupts vein development in Arabidopsis, affecting the transport of nutrients and photosynthetic products, which ultimately influences plant growth under cadmium stress.

#### Chloroplast retrograde signaling regulates seedling response to CdCl_2_ stress

**Fig. 9:**
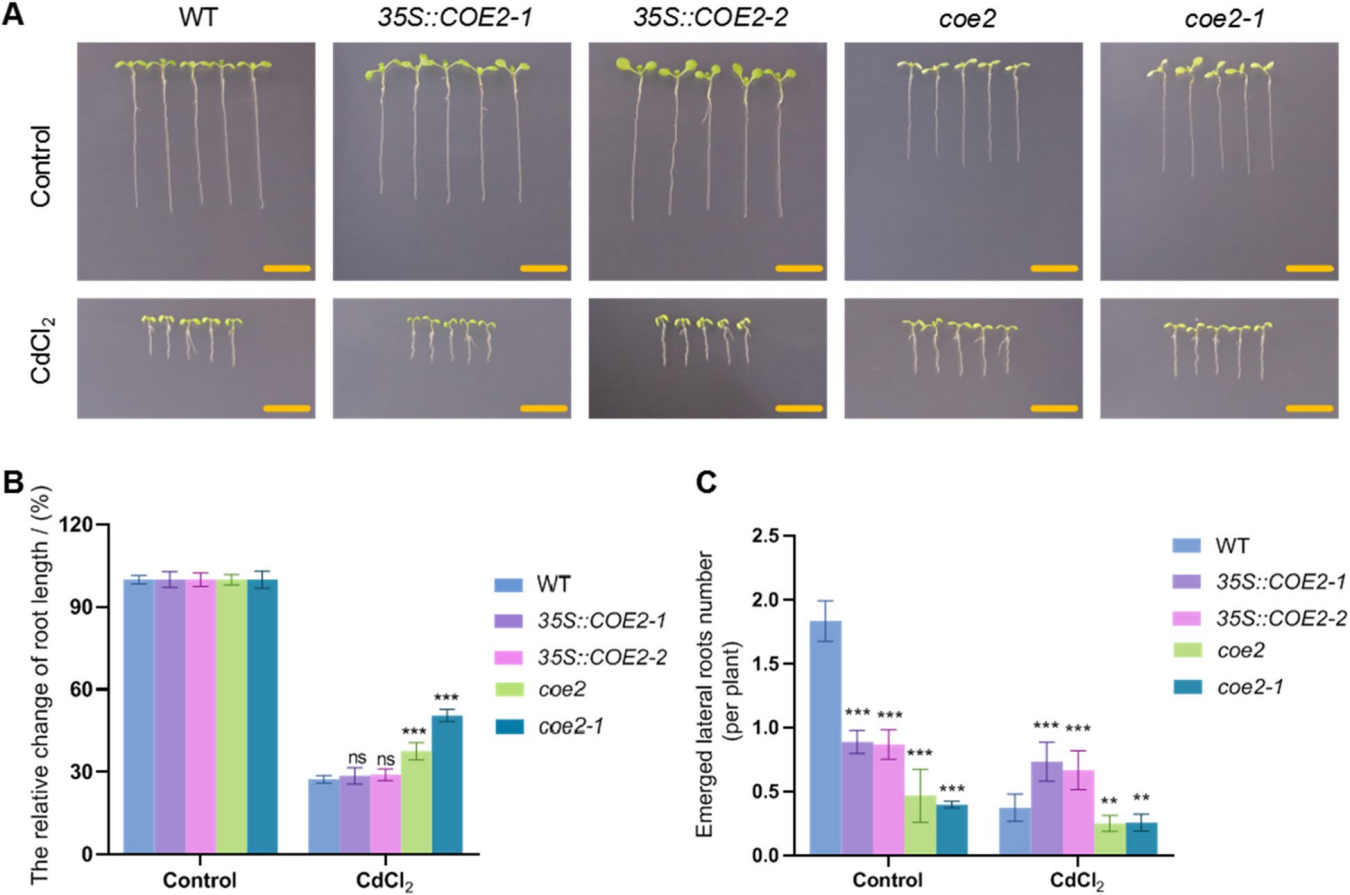
Growth and developmental phenotypes of WT, *35S::COE2-1*, *35S::COE2-2*, *coe2* and *coe2-1* seedlings under normal and cadmium stress conditions. **A** The growth and developmental phenotypes of WT, *35S::COE2-1*, *35S::COE2-2*, *coe2* and *coe2-1* seedlings after 7 days of growth on normal and 1/2MS medium supplemented with CdCl_2_. Scale bar = 1 cm. **B** The relative percentage of changes in seedling root length; The error line represents the standard deviation, and three biological replicates (*n* = 5) were performed to determine the significant difference between wild-type and overexpressing plants through one-way ANOVA, *** *p* < 0.001. **C** Statistical analysis of lateral root numbers in seedlings; Perform 3 biological replicates (*n* = 5) and determine the significant differences between WT and overexpressing plants through one-way ANOVA, *** *p* < 0.001

The above analysis indicates that CdCl_2_ affects chloroplast function, thereby influencing plant growth and development by regulating the expression of chloroplast proteins such as J3 and PsbO1. Studies have shown that chloroplast retrograde signaling regulates chloroplast development(El Rasafi et al. 2022). Our previous research identified CHLOROPHYLL A/B-BINDING OVEREXPRESSION 2 (COE2) involvement in regulating chloroplast retrograde signaling(Wu et al. 2022). To explore the role of chloroplast retrograde signaling in regulating chloroplast development under CdCl_2_ stress, we analyzed the effects of CdCl_2_ on the growth of *35S::COE2-1*, *35S::COE2-2*, *coe2*, and *coe2-1* seedlings. Results showed that compared to control, CdCl_2_ treatment significantly affected root length and lateral root development in WT, *35S::COE2-1*, *35S::COE2-2*, *coe2*, and *coe2-1* seedlings (Fig. 9). Under control conditions, root length of *35S::COE2-1* and *35S::COE2-2* seedlings did not significantly differ from WT, while *coe2* and *coe2-1* seedlings showed significantly shorter root lengths than WT (Fig. 9 A and B). In contrast, under CdCl_2_ conditions, root lengths of *35S::COE2-1* and *35S::COE2-2* seedlings did not significantly differ from WT, while root lengths of *coe2* and *coe2-1* seedlings were significantly longer than WT (Fig. 9 A and B). Compared to control conditions, CdCl_2_ treatment significantly reduced lateral root numbers in WT, *35S::COE2-1*, *35S::COE2-2*, *coe2*, and *coe2-1* seedlings. Under control conditions, lateral root numbers in *35S::COE2-1*, *35S::COE2-2*, *coe2*, and *coe2-1* seedlings were significantly lower than WT (Fig. 9 A and C). After CdCl_2_ treatment, lateral root numbers in *35S::COE2-1* and *35S::COE2-2* seedlings were significantly higher than WT (Fig. 9 A and C). These results indicate reduced sensitivity of *coe2* and *coe2-1* seedlings to CdCl_2_ stress compared to WT.

#### COE2 is required for regulating the chloroplast function in response to CdCl_2_ stress

We assessed chlorophyll fluorescence in seedlings of *35S::COE2-1*, *35S::COE2-2*, *coe2*, and *coe2-1* under normal conditions. The results indicated that, except for *coe2* and *coe2-1* seedlings, the Fv/Fm levels were lower than those in WT seedlings (Fig.S8 A, B). Notably, the Fv/Fm levels in *35S::COE2-1* and *35S::COE2-2* seedlings were similar to WT levels (Fig.S8 A, B). Under CdCl_2_ stress, Fv/Fm levels significantly decreased in WT seedlings compared to control conditions (Fig.S8A, B). While the Fv/Fm levels also dropped in *35S::COE2-1* and *35S::COE2-2* seedlings, the reduction was less severe than in WT plants (Fig.S8 A, B). Interestingly, in *coe2* and *coe2-1* seedlings, Fv/Fm levels not only remained stable but actually increased under CdCl_2_ stress (Fig.S8 A, B). These findings suggest that CdCl_2_ treatment activates COE2-dependent signaling in chloroplasts, enhancing their stress response.

**Fig. 10:**
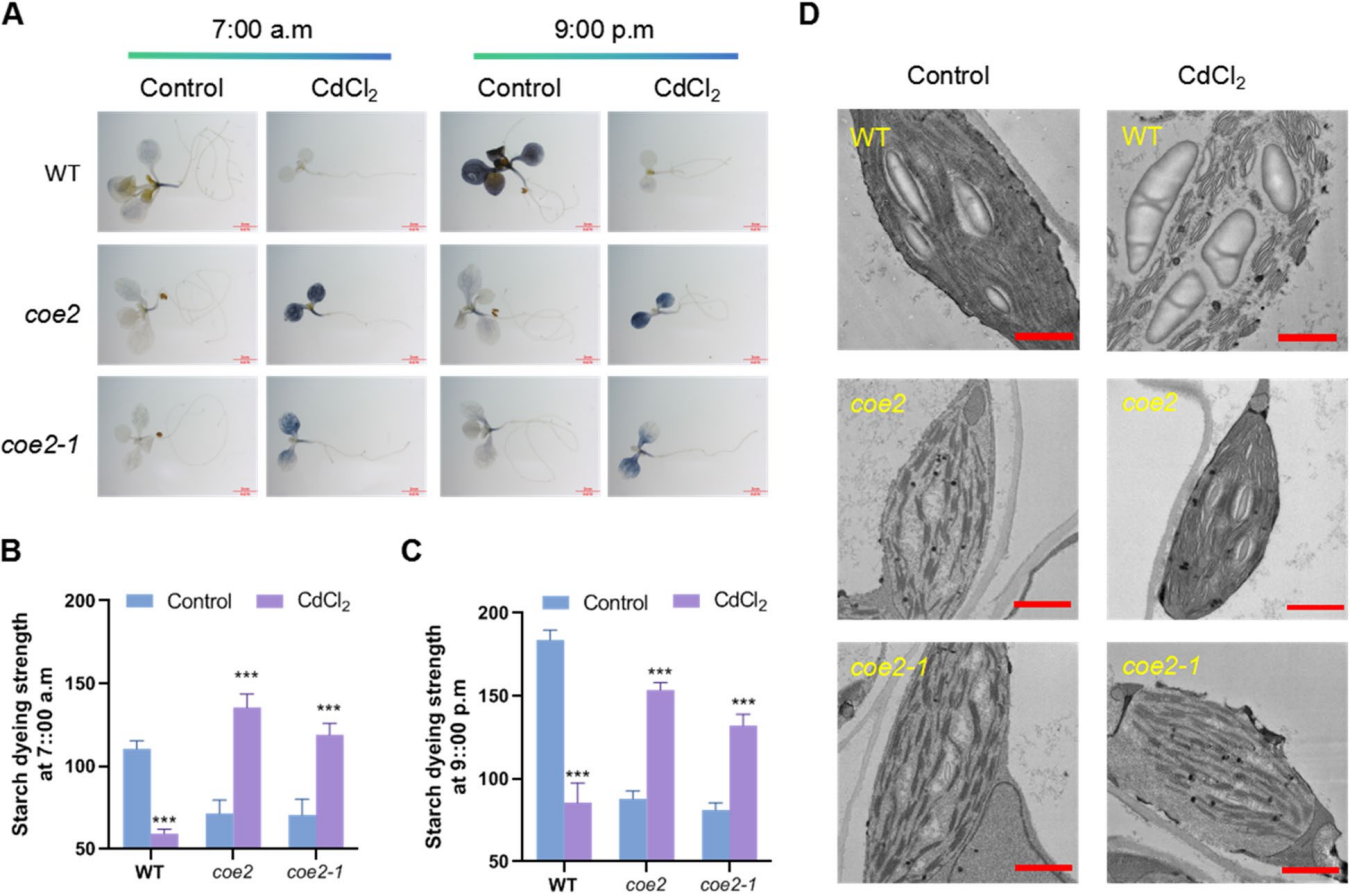
Analysis of the effects of CdCl_2_ treatment on the synthesis and metabolism of starch in WT, *coe2*, and *coe2-1* seedlings. **A** Analyze the effects of CdCl_2_ on starch synthesis and metabolism in WT, *coe2*, and *coe2-1* seedlings using I_2_/KI staining method. All seedlings were grown on control and CdCl_2_ media for 7 days, and the materials were taken at 7 am and 9 pm. After decolorization with 70% alcohol, starch staining was detected using a stereomicroscope. Scale bar = 2 mm. **B** Statistics on starch staining intensity of WT, *coe2*, and *coe2-1* seedlings at 7 am; The error line represents the standard deviation, and the significance of the difference under CdCl_2_ conditions compared to normal conditions was analyzed using Student’s t-test (*n* = 3), *** *p* < 0.001. **C** At 9 pm, the starch staining intensity of WT, *coe2*, and *coe2-1* seedlings was statistically analyzed. The error line represents the standard deviation. Student’s t-test was used to analyze the significance of the difference between CdCl_2_ conditions and normal conditions (*n* = 3), *** *p* < 0.001, ** *p* < 0.01. **D** Using transmission electron microscopy to detect and analyze the chloroplasts of WT, *coe2*, and *coe2-1* seedlings under control and CdCl_2_ conditions. The red line represents the scale bar, which is 1 μm

We further examined starch metabolism in WT, *coe2*, and *coe2-1* seedlings under both normal and CdCl_2_ conditions. Under control conditions, starch staining was weaker in *coe2* and *coe2-1* seedlings compared to WT (Fig. 10). However, following CdCl_2_ treatment, starch staining was significantly stronger in *coe2* and *coe2-1* seedlings than in WT (Fig. 10). Additionally, starch levels in WT, *coe2*, and *coe2-1* seedlings all decreased overnight under normal conditions (Fig. 10). Under CdCl_2_ stress, WT seedlings showed no significant change in starch metabolism between day and night, whereas starch accumulation in *coe2* and *coe2-1* seedlings remained elevated both in the evening and the following morning (Fig. 10).

Using transmission electron microscopy, we analyzed chloroplast ultrastructure in WT, *coe2*, and *coe2-1* seedlings under both normal and CdCl_2_ conditions. CdCl_2_ treatment disrupted the thylakoid membrane structure in WT seedlings, resulting in loose grana stacking (Fig. 10). In contrast, *coe2* and *coe2-1* seedlings exhibited reduced thylakoid membrane structure and grana compared to WT under normal conditions (Fig. 10). Remarkably, under CdCl_2_ stress, the thylakoid membranes in *coe2* and *coe2-1* seedlings remained relatively intact (Fig. 10).

Given that J3 and PsbO1 are chloroplast-targeted proteins regulated by retrograde signaling, we examined their expression in *coe2* and *coe2-1* seedlings under CdCl_2_ stress. In WT seedlings, *J3* and *PsbO1* expression did not significantly change under CdCl_2_ conditions compared to controls (Fig.S9). Conversely, in *coe2* and *coe2-1* seedlings, the expression levels of *J3* and *PsbO1* were significantly higher under both normal and CdCl_2_ conditions compared to WT (Fig.S9). These results suggest that *coe2* and *coe2-1* seedlings respond to CdCl_2_ stress by upregulating *J3* and *PsbO1* gene expression.

#### COE2 dependant signaling is involved in regulating the quality-control of chloroplast

Our observations of leaf vein development in WT, *coe2*, and *coe2-1* seedlings under both control and CdCl_2_ conditions revealed that CdCl_2_ significantly affected leaf vein development in WT seedlings (Fig.S10 A and B). In contrast, *coe2* and *coe2-1* seedlings showed no significant differences in leaf vein development between control and CdCl_2_ conditions, and their leaf vein conduits did not exhibit noticeable fractures or loosening (Fig.S10 A and B).

CdCl_2_ stress disrupted the chloroplast thylakoid membrane structure and affected starch metabolism in WT seedlings. However, in *35S::J3-1*, *35S::J3-2*, *35S::PSBO1-1*, *35S::PSBO1-2*, *coe2*, and *coe2-1* seedlings, chloroplast structures remained intact under CdCl_2_ stress. This suggests that J3, PSBO1, and COE2 may play roles in protecting chloroplast structure and regulating starch metabolism. Chloroplast starch metabolism is crucial for seedling metabolic responses, while damage to the chloroplast thylakoid membrane is a typical stress response. CdCl_2_-induced chloroplast damage and starch metabolism disturbances in WT seedlings suggest that cells may respond to CdCl_2_ stress by accelerating the degradation of damaged chloroplasts and weakening energy metabolism cycles.

Previous studies have shown that the plant U-box4 (PUB4) E3 ubiquitin ligase regulates autophagy of chloroplasts damaged by reactive oxygen species (ROS) in FERROCHELATASE2 (FC2) mutant seedlings (Woodson et al. 2015). Additionally, FC1 is involved in regulating retrograde signaling in chloroplasts(Woodson et al. 2011). To explore the potential role of retrograde signaling in chloroplast function under CdCl_2_ stress, we analyzed the expression of *FC1*, *U-BOX 4* (*PUB4*), and *U-BOX 3* (*PUB3*) in WT, *35S::PSBO1-1*, *35S::PSBO1-2*, *35S::J3-1*, *35S::J3-2*, *coe2*, and *coe2-1* seedlings under CdCl_2_ stress. *PUB3* was included to observe if genes with similar functions are also affected by CdCl_2_ stress.

The results showed significant upregulation of *FC1*, *PUB3*, and *PUB4* expression in all seedlings under CdCl_2_ stress compared to control conditions. However, the expression levels of these genes in *35S::PSBO1-1*, *35S::PSBO1-2*, *35S::J3-1*, *35S::J3-2*, *coe2*, and *coe2-1* seedlings were lower than in WT under CdCl_2_ stress (Fig.S11). Although *PUB3* expression was induced by CdCl_2_ stress, the increase was less pronounced than *PUB4*, indicating that PUB4 may be the primary regulatory factor for degrading damaged chloroplasts under stress conditions.

## Discussion

In previous studies, we found that genes specifically expressed in the vascular bundle tissues of Arabidopsis seedling veins are involved in regulating heavy metal stress(Liu et al. 2022c). El-Okkiah et al. observed changes in the number and structure of vessels in stems and roots of plants under cadmium treatment, indicating that cadmium affects the development of plant vascular tissues(El-Okkiah et al. 2022). These results suggest that vascular tissues are the primary sites where plants respond to cadmium stress. To explore the mechanisms by which Arabidopsis seedlings regulate their response to cadmium stress, we analyzed published RNA-seq data induced by heavy metal cadmium and screened for differentially expressed genes. Through GO enrichment analysis, we found that cadmium-induced genes were enriched in GO terms related to “detoxification” and genes specifically expressed in companion cells of the phloem enriched in GO terms related to “response to metal ion,” indicating their involvement in similar biological processes in response to metal ion stress (Fig. 1).

To study the effects of cadmium stress on vascular plant growth and development, we identified five genes specifically expressed in the vascular tissue through cellular expression visualization: *AHP1*, *DOF2.4*, *NHL1*, *CDF4*, and *ATARFB1A*. Through qPCR analysis and GUS reporter gene analysis (Fig. 2), we found that CdCl_2_ treatment induces the expression of these genes. Therefore, we generated overexpression plants for these genes to study their role in regulating plant responses to CdCl_2_ stress. By observing the developmental status of the roots (Fig. 3), vascular tissue development of leaf veins (Fig. 4), and photosynthetic function (Fig. 5) in Arabidopsis seedlings overexpressing these genes, we found that overexpression of these genes specifically expressed in vascular tissues enhances tolerance to cadmium stress. These results suggest that enhancing vascular bundle development benefits Arabidopsis seedlings in resisting cadmium stress.

### J3 and PsbO1 Chloroplast proteins involved in Arabidopsis response to cadmium

Chu et al. found that cadmium stress disrupts electron transfer and energy distribution between PSII and PSI, affecting photosynthesis in plants (Chu et al. 2018). Paunov et al. found that cadmium disrupts photosynthetic electron transfer processes, thereby inhibiting the efficiency of energy conversion in photosystem II (Paunov et al. 2018). Xue et al. found that cadmium stress leads to a decrease in chlorophyll content in soybean seedling leaves and a decrease in CO_2_ assimilation capacity in mesophyll cells, thereby inhibiting electron transfer in chloroplasts (Xue et al. 2014). Through our analysis, we found that cadmium stress affects chloroplast photosynthetic function, and overexpression of vascular-specific genes can mitigate the impact of CdCl_2_ on photosynthetic function in Arabidopsis seedlings, suggesting that increased tolerance to CdCl_2_ stress in overexpressing seedlings may result in relatively stable photosynthetic function. To investigate whether maintaining photosynthetic function in leaf vein microtubules contributes to increased seedling resistance to CdCl_2_ stress, we identified two chloroplast related genes, *J3* and *PsbO1*, highly expressed in vascular bundle tissues (Takahashi et al. 2017; Zhou et al. 2018). qPCR analysis revealed that the expression of these two genes is induced by CdCl_2_ stress. We generated overexpression plants for these two genes and analyzed the tolerance of overexpressing seedlings to CdCl_2_ stress. The results showed that compared to WT, *35S::J3* and *35S::PSBO1* seedlings exhibited significantly improved root development (Fig. 6), chlorophyll fluorescence (Fig.S7), stability of chloroplast thylakoid membrane structure (Fig. 7), and vascular bundle development of leaf veins under cadmium stress (Fig. 8), particularly significant improvement in the vascular bundle development of leaf veins (Fig. 8). These results indicate that maintaining photosynthetic function in leaf vein tissues or leaves may play a protective role in leaf vein development under cadmium stress. This maintenance of microtubule development in leaf veins may also increase seedling resistance to cadmium stress.

### Impact of cadmium stress on leaf vein development

Our study revealed that cadmium stress adversely affects leaf vein development in WT seedlings, causing loosening and breakage of the vascular bundles and conduits (Fig. 8 and Fig.S10). In contrast, leaf vein development in *35S::J3-1*, *35S::J3-2*, *35S::PSBO1-1*, *35S::PSBO1-2*, *coe2*, and *coe2-1* seedlings remained unaffected under the same conditions (Fig. 8 and Fig.S10). This suggests that cadmium stress inhibits leaf vein development in WT seedlings, reducing the transport of water and nutrients, which could hinder overall plant growth and development. However, the transgenic and mutant lines appear to protect vascular tissue development, likely by enhancing photosynthetic function, thus maintaining the transport of water and nutrients and increasing tolerance to cadmium stress.

Leaf veins play a crucial role in transporting water and dissolved nutrients from the roots and stems to the leaves and distributing photosynthetic products throughout the plant. Previous research supports this understanding. Notaguchi et al. noted that the plant vascular system serves not only as a material conduit but also as a long-distance communication pathway, helping plants adapt to environmental changes (Notaguchi and Okamoto 2015). Scarpella et al. described feedback loops in auxin transport within leaf vascular tissues that regulate their transport (Scarpella et al. 2010). Huang et al. found a positive correlation between the area of mesophyll and phloem in rice leaves and photosynthesis (Scarpella et al. 2010). Our investigation into leaf vein development in WT, *35S::PSBO1*, *35S::J3*, *coe2*, and *coe2-1* plants, combined with analysis of their photosynthetic processes and products, demonstrated that cadmium stress in Arabidopsis inhibits leaf vein development. This inhibition disrupts nutrient transport within leaf veins, consequently affecting seedling growth.

### Cadmium stress causes chloroplast damage and quality control

Studies have shown that when plants face environmental stresses such as drought, nutrient deficiency, oxidative stress, salt, and nitrogen deficiency, they initiate catabolic processes and undergo autophagy to remove harmful components within the body (Doelling et al. 2002; Xiong et al. 2007). Our results indicate that cadmium stress causes damage to chloroplast structures. Research has found that damaged chloroplasts are degraded through a PUB4-mediated ubiquitination pathway, achieving chloroplast quality control within cells (Woodson et al. 2015). Therefore, we hypothesize that cadmium stress in plants also leads to chloroplast damage and degradation.

Firstly, we examined starch synthesis and metabolism under cadmium stress in WT, *35S::J3-1*, *35S::J3-2*, *35S::PSBO1-1*, *35S::PSBO1-2*, *coe2*, and *coe2-1*. The results showed that cadmium stress affects starch synthesis and metabolism in these seedlings, indicating that chloroplast function is compromised (Fig. 7 and Fig. 10). Then, we detected the relative expression levels of key genes involved in chloroplast retrograde signaling and ubiquitin-mediated degradation, *FC1* and *PUB4*, in WT, *35S::J3-1*, *35S::J3-2*, *35S::PSBO1-1*, *35S::PSBO1-2*, *coe2*, and *coe2-1*. Under CdCl_2_ conditions, we found that *FC1* and *PUB4* were significantly upregulated in all these seedlings, with expression levels in WT being higher than in the other seedlings (Fig.S11). These results reveal two potential mechanisms in Arabidopsis seedlings under cadmium stress: (1) Cadmium stress causes chloroplast damage and induces the degradation of damaged chloroplasts. Damaged chloroplasts trigger plastid retrograde signaling, which then regulates genes related to chloroplast development and quality control, such as *J3*, *PSBO1*, *FC1*, and *PUB4*, through COE2-mediated signaling pathways, to maintain chloroplast function. The maintenance of chloroplast photosynthetic function provides energy for the development of vascular tissues, promoting leaf vein development and seedling growth. (2) As vascular tissue is the main transport channel for cadmium absorption and translocation in plants, cadmium absorption by seedlings initially causes damage to cells in vascular tissues. Therefore, genes specifically expressed in vascular tissues, such as *HIPP36*, directly participate in regulating the response to cadmium stress. Additionally, other genes involved in regulating vascular tissue development enhance seedling resistance to cadmium stress by promoting vascular tissue development.

## Conclusion

**Fig. 11:**
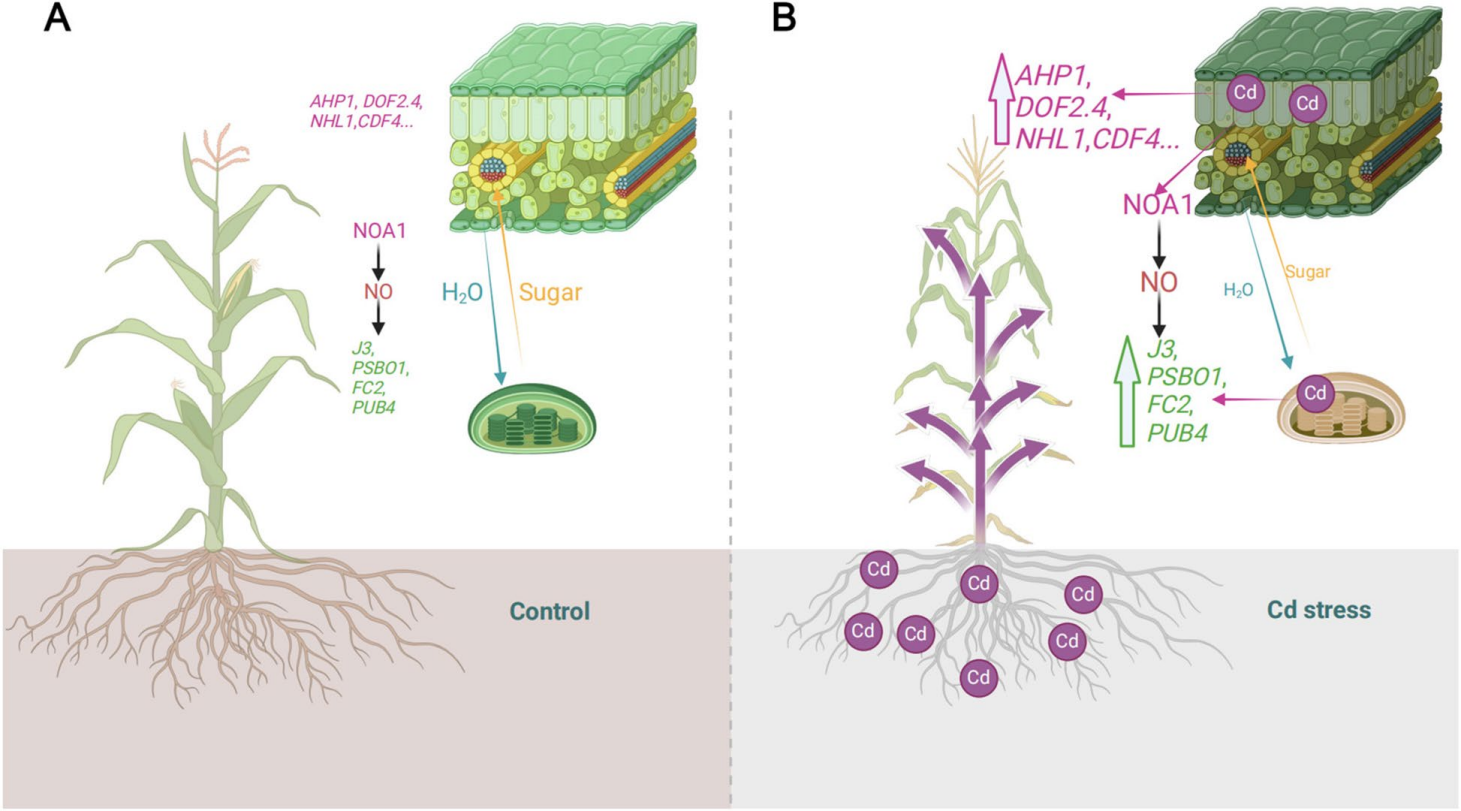
A schematic representation elucidating the mechanism by which NOA1-mediated nitric oxide (NO) signaling modulates plant adaptive responses to cadmium (Cd) stress. **A** The left panel of the diagram illustrates seedlings cultivated under optimal, non-stress conditions. Under such circumstances, the coordinated expression of genes involved in the development of vascular tissues within leaf veins, in conjunction with those controlling chloroplast biogenesis and function, facilitates the establishment of well-structured vascular networks. These networks are crucial for the efficient transport of water and photosynthates (e.g., sugars), thereby supporting sustained plant growth and developmental processes. **B** In stark contrast, the right panel highlights the detrimental effects imposed by Cd stress, manifesting as pronounced suppression of both root system development and the expansion of aerial tissues, particularly leaves. Cd exposure induces a transcriptional upregulation of genes involved in vascular tissue formation, likely serving as an adaptive response aimed at maintaining the structural and functional integrity of these tissues under toxic conditions. Simultaneously, NOA1-mediated NO signaling amplifies the expression of genes essential for chloroplast maintenance, thereby ensuring the preservation of chloroplast homeostasis and accelerating the quality control mechanisms responsible for the repair of damaged organelles

In this study, our findings demonstrate that cadmium stress significantly disrupts photosynthesis and vascular development in wild-type plants, leading to growth defects and tissue damage. However, plants overexpressing some key vascular-specific genes, *AHP1*, *DOF2.4*, *NHL1*, *CDF4*, and *ATARFB1A*, showed increased resilience to cadmium stress. The enhanced expression of chloroplast-associated proteins PSBO1 and J3 was particularly effective in preserving the integrity of thylakoid membranes, thereby improving chlorophyll fluorescence and promoting starch metabolism. This indicates that chloroplast retrograde signaling, mediated by COE2, plays a central role in protecting chloroplasts from cadmium damage by regulating the expression of *J3* and *PSBO1*. This protection extends to vascular tissues, supporting nutrient transport and enhancing overall stress resilience. A key insight from this study is the connection between chloroplast function and vascular development under cadmium stress. The vascular system, especially the leaf veins, plays a vital role in distributing water and nutrients throughout the plant. Disruption of this system can severely impair growth. Our results suggest that the overexpression of vascular-specific genes helps protect leaf vein development, ensuring the uninterrupted transport of essential nutrients even in the presence of cadmium. Additionally, cadmium stress triggers the degradation of damaged chloroplasts via a PUB4-mediated chloroplast quality control. The upregulation of *PUB4* and *FC1* during cadmium stress supports their critical role in maintaining chloroplast integrity and energy balance, helping plants cope with environmental stress. In conclusion, this study provides valuable insights into the genetic regulation of Arabidopsis’ response to cadmium stress, emphasizing the importance of both vascular tissue development and chloroplast protection (Fig. 11). The identification of specific genes that increase tolerance to cadmium offers promising targets for improving heavy metal resistance in crops. By safeguarding chloroplast function and supporting vascular system integrity, these genes enable plants to maintain growth and development under challenging conditions. As environmental challenges continue to mount, a deeper understanding of these protective mechanisms will be essential for developing plants better suited to survive and thrive under harsh conditions.

## Materials and methods

### Cultivation of Arabidopsis

The seeds used in this study including of WT, *35S::COE2-1*, *35S::COE2-2*, *coe2*, *coe2-1*, *35S::PSBO1-1*, *35S::PSBO1-2*, *35S::J3-1*, *35S::J3-2*, *DOF2.4pro::GUS*, *CDF4pro::GUS*, *ATARFB1Apro::GUS*, *35S::AHP1*, *35S::DOF2.4*, *35S::NHL1*, *35S::CDF4*, and *35S::ATARFB1A.* Begin by placing a suitable amount of Arabidopsis seeds into a 1.5 mL EP tube. In a laminar flow hood, wash the seeds with 75% ethanol for 4 min, followed by 10% sodium hypochlorite disinfectant for 10 min. Rinse the seeds 5–7 times with sterile water to remove any remaining disinfectant. Sow the disinfected seeds onto the culture medium and stratify them at 4 °C for 2–3 days. After stratification, place the plates in the growth room (22 °C-25 °C, 16-h light/8-h dark cycle) for 7 days. Considering that the development of lateral roots not only involves the number produced, but also relates to the length of their development. Therefore, we counted the number of lateral roots that can be directly observed in the seedling root system and used ImageJ software to measure the length of lateral roots.

### Extraction of Arabidopsis DNA

*Arabidopsis* DNA was extracted using the CTAB (cetyltriethylammnonium bromide) method. First, weigh approximately 0.1 g of Arabidopsis tissue and grind it into a powder with liquid nitrogen in a mortar. Add 650 μL of 65 °C preheated CTAB to the mortar, then transfer the mixture to a 1.5 mL EP tube. Vortex to mix, add an equal volume of chloroform, gently mix, and centrifuge at 12,000 g for 10 min. Transfer the supernatant to a clean 1.5 mL EP tube, add an equal volume of isopropanol, gently mix, and let it stand at room temperature for 10 min. Transfer the mixture to a DNA adsorption column, centrifuge at 12,000 g for 5 min, and discard the liquid in the collection tube. Add 700 μL of 70% ethanol to the collection tube, centrifuge at 12,000 g for 3 min, and discard the filtrate. Repeat this step once more. Place the adsorption column back into the collection tube and centrifuge at 12,000 g for 5 min to remove excess ethanol. To complete the process, place the DNA adsorption column into a clean 1.5 mL EP tube, leave the lid open, and let it stand for 5–8 min to allow the ethanol to evaporate completely. Add 30–50 μL of preheated sterile water (65 °C) to the adsorption column, let it stand at room temperature for 3–5 min, and centrifuge at 12,000 g for 5 min. For increased DNA concentration, elute the DNA solution through the adsorption column again, let it stand for 2 min, and centrifuge once more to collect the DNA.

### Extraction of Arabidopsis RNA

Total RNAs were extracted with the E.Z.N.A. Plant RNA Kit (Omega Bio-Tek), according to the manufacturer’s instructions. Place approximately 0.1 g of Arabidopsis tissue sample into a 2 mL EP tube and freeze in liquid nitrogen for 5 min before grinding into a fine powder. Add 500 μL of RB Buffer to the powder and thoroughly mix using a vortex. Transfer the mixture onto a gDNA spin column placed in a 2 mL collection tube. Centrifuge at 14,000 g for 5 min at room temperature, transfer the filtrate to a new EP tube, and add 0.5 volumes of anhydrous ethanol, mixing well. Transfer 700 μL of the mixed solution into a HiBind RNA Mini filter column, centrifuge at 12,000 g for 1 min at room temperature, and discard the filtrate. Add 400 μL of RWF Wash Buffer to the column, centrifuge at 10,000 g for 30 s, and discard the filtrate. Follow with 500 μL of RNA Wash Buffer II, centrifuge at 10,000 g for 30 s, and discard the filtrate. Centrifuge at 12,000 g for 5 min to remove residual ethanol from the HiBind RNA Mini filter column. Finally, add 30–50 μL of preheated DEPC water (65 °C) to the HiBind RNA Mini filter column, incubate at room temperature for 3–5 min, and centrifuge at 12,000 g for 2 min. Collect the RNA solution for subsequent RNA reverse transcription experiments.

### Synthesis of Arabidopsis cDNA

To remove residual genomic DNA from the extracted total RNA, add 0.1 ng to 1 μg of extracted RNA, 1 μL of gDNA Purge, and RNase-Free Water to a 200 μL EP tube to achieve a total volume of 10 μL. Incubate at 42 °C for 5 min in a metal bath, then place on ice. Add 10 μL of 2 × NovoScript Plus 1st Strand cDNA Synthesis SuperMix (Novoprotein, Suzhou, China) to the 10 μL reaction mixture, achieving a total volume of 20 μL. Gently mix, centrifuge, and incubate in a PCR machine at 50 °C for 15 min, followed by 75 °C for 5 min to terminate the reaction. The resulting product can be directly used for qRT-PCR or stored at -20 °C.

### Gene expression analysis by qRT-PCR

For gene expression analysis, quantitative real-time polymerase chain reaction (qRT-PCR) experiments were conducted using SYBR Green I fluorescent dye with the gene specific primers (Table S4). The PCR mix, consisting of 10 μL of 2 × Novo Start SYBR qPCR Super Mix Plus (Novoprotein, Suzhou, China), 1.5 μL each of forward and reverse primers, 1.5 μL of template cDNA, and RNase-Free Water to a total volume of 20 μL, was added to a dedicated qRT-PCR 96-well plate. After centrifugation and mixing, the plate was placed into a qTOWER3G instrument for real-time PCR detection, following a thermal profile of initial denaturation at 95 °C for 1 min, followed by 30 cycles of amplification. Relative gene expression levels were calculated based on Ct values obtained from the reaction.

### Vector construction

To construct vectors, the target gene fragment was amplified using KOD OneTM PCR Master Mix enzyme (TOYOBO, Osaka, Japan) with Arabidopsis wild-type (WT) cDNA and genomic DNA (gDNA) as templates. Purification of the correct fragments was achieved using the Thermo Scientific GeneJET Gel Extraction Kit (Thermo Scientific). The purified target gene fragment and the binary expression vector pCAMBIA2300 were digested using restriction enzymes *Xba*I and *BamH*I, followed by gel extraction of the digested products. The target gene fragment was ligated with the linearized vector plasmid, and the resulting recombinant plasmid was transformed into *Escherichia coli* DH5α Competent Cells. Positive recombinant plasmids were selected and further transformed into *Agrobacterium tumefaciens* GV3103 Chemically Competent Cells.

### Identification of transgenic Arabidopsis plants

Wild-type (WT) Arabidopsis seedlings were used as the recipient material for Agrobacterium-mediated transformation via the floral dip method. After maturation of Arabidopsis seeds following *Agrobacterium* infection, seeds were harvested. Positive seedlings were selected by surface sterilization of harvested seeds, sow to 1/2 MS media containing appropriate antibiotics from the vector, and subsequent cultivation. Successfully identified plants were cultivated to the T3 generation to obtain stable homozygous transgenic Arabidopsis plants for subsequent experiments.

### Chlorophyll fluorescence detection

Arabidopsis samples were prepared with cotyledons and true leaves facing up on a petri dish, covered with foil, and kept in darkness for 30 min. Chlorophyll fluorescence was measured using a fluorescence imager to observe leaf material changes. Data analysis, including Fv/Fm calculation, was performed using ImagingWin software for statistical analysis.

### GUS Staining

GUS staining involved preparing a solution according to the X-gluc stock solution base ratio and immersing samples in the staining solution in centrifuge tubes. Vacuum was applied to ensure complete immersion, followed by incubation in the dark at 37 °C until blue staining indicated successful GUS activity. Destaining was achieved by transferring samples to 70% ethanol at 65 °C until leaves became colorless and transparent, allowing visualization and photography under a stereomicroscope.

### Starch staining

For starch staining, Arabidopsis samples were destained with 70% ethanol at 65 °C until leaves became white and transparent. Staining was performed using an I_2_/KI solution, followed by rinsing with ddH_2_O for observation and photography under a stereomicroscope. The signal intensity of starch staining was analyzed using ImageJ. The statistical analysis was performed with Student’s t-test on the obtained signal strength data and calculate the accumulation level of starch in leaf samples.

### Vein fixation and transparency

Vein fixation and transparency required a specialized solution. Samples were treated with 70% ethanol and subjected to destaining at 60 °C, followed by fixation with a prepared vein fixation solution. After fixation, samples were stored for observation under a phase contrast microscope.

### Preparation of ultrathin sections and transmission *electron* microscope (TEM) Observation

To prepare the embedding agent, EPon 812 (24 g), Dodecenylsuccinic anhydride (DDSA) (9 g), and Methyl Nadic anhydride (MNA) (15 g) were used. Plant samples of 1 mm × 2 mm were immersed in a fixative solution (5% glutaraldehyde + 4% paraformaldehyde) under vacuum, ensuring complete immersion of leaf sections, and left overnight for fixation. Following this, samples were rinsed three times with 0.1 M PBS (pH 7.1) for 15 min each. Subsequently, the samples were fixed with 1% osmium tetroxide in 0.1 M PBS for 3–4 h and rinsed again three times with 0.1 M PBS (pH 7.1) for 15 min each. The dehydration process involved sequential treatment with 30%, 50%, 70%, and 90% ethanol, followed by 90% and pure acetone. The samples were then infiltrated with a gradient of embedding agent in acetone solutions (25%, 50%, 75%, and 100%) for 6–12 h each. Pre-embedding was conducted by adding 30% Dimethyl Phthalate (DMP) to the embedding agent in the embedding dish, with approximately 100 μL of embedding agent added to the samples. Samples were positioned as required and additional embedding agent was added until the dish was full, taking care to avoid bubbles. Polymerization was carried out at 37 °C for 12 h, 45 °C for 12 h, and 60 °C for 48 h. Once polymerization was complete, the samples were stored at room temperature. Ultrathin sections were then cut to the desired thickness using an ultramicrotome and observed under a transmission electron microscope (TEM).

## Supplementary Information

Additional file 1: Fig.S1 Analysis of relative expression levels of cadmium stress-induced genes under cadmium stress treatment and normal conditions. Fig.S2 Analysis of the developmental status of cotyledon veins in young seedlings under normal and cadmium stress conditions. Fig.S3 Visualization and relative expression analysis of genes specifically expressed in vascular tissues. Fig.S4 Detection of expression levels of *AHP1*, *DOF2.4*, *CDF4*, *NHL1*, and *ATARFB1A* in corresponding transgenic plants. Fig.S5 Visualization and relative expression analysis of *PSBO1* and *J3 *genes in cells. Fig.S6 Detection of expression levels of *PSBO1* and *J3* in corresponding transgenic plants. Fig.S7 Chlorophyll fluorescence measurement and statistical analysis of WT, *35S::PSBO1 *and *35S::J3* seedlings. Fig.S8 Chlorophyll fluorescence measurement and statistical analysis of WT, *35S::COE2-1*, *35S::COE2-2*, *coe2*, and *coe2-1*seedlings. Fig.S9 Detection of expression levels of *J3 *and *PSBO1* in WT, *coe2*, and* coe2-1* seedlings. Fig.S10 Analysis of the effects of CdCl_2_ treatment on the development of leaf vein tissues in WT, *coe2*, and *coe2-1* seedlings. Fig.S11 Effects of CdCl_2_ treatment on the expression of*FC1*, *PUB3*, and *PUB4*.

Additional file 2: Table S1. The differentially expressed genes between Cd treatment and control. Table S2. GO analysis of DEGs between Cd treatment and control. Table S3. GO analysis of DEGs in companion cell.

## Data Availability

All data had been supplied in supplementary information.

## Abbreviations

DEGs: Differentially expressed genes
GO: Gene Ontology
RNA-seq: RNA sequencing
RT-qPCR: Reverse transcription quantitative PCR
scRNA-seq: Single cell RNA sequencing
TEM: Transmission electron microscope
DMP: Dimethyl Phthalate
DEGs: Differentially expressed gene
