# Supplementary material for: The crucial role of vascular tissues and COE2-mediated retrograde signaling in mitigating cadmium stress in Arabidopsis thaliana
Source: Stress Biol. 2025 Feb 6;5(1):10. doi: 10.1007/s44154-024-00196-4 (PMC11799468; doi:10.1007/s44154-024-00196-4)
Supplement: Supplementary file 1 — Additional file 1: Fig.S1 Analysis of relative expression levels of cadmium stress-induced genes under cadmium stress treatment and normal conditions. Fig.S2 Analysis of the developmental status of cotyledon veins in young seedlings under normal and cadmium stress conditions. Fig.S3 Visualization and relative expression analysis of genes specifically expressed in vascular tissues. Fig.S4 Detection of expression levels of AHP1, DOF2.4, CDF4, NHL1, and ATARFB1A in corresponding transgenic plants. Fig.S5 Visualization and relative expression analysis of PSBO1 and J3 genes in cells. Fig.S6 Detection of expression levels of PSBO1 and J3 in corresponding transgenic plants. Fig.S7 Chlorophyll fluorescence measurement and statistical analysis of WT, 35S::PSBO1 and 35S::J3 seedlings. Fig.S8 Chlorophyll fluorescence measurement and statistical analysis of WT, 35S::COE2-1, 35S::COE2-2, coe2, and coe2-1seedlings. Fig.S9 Detection of expression levels of J3 and PSBO1 in WT, coe2, and coe2-1 seedlings. Fig.S10 Analysis of the effects of CdCl2 treatment on the development of leaf vein tissues in WT, coe2, and coe2-1 seedlings. Fig.S11 Effects of CdCl2 treatment on the expression ofFC1, PUB3, and PUB4. [file 44154_2024_196_MOESM1_ESM.docx]

**Supplementary Information**

**Fig.S1** Analysis of relative expression levels of cadmium stress-induced genes under cadmium stress treatment and normal conditions.

To compare and analyze the relative expression levels of cadmium stress-induced genes under cadmium stress and normal conditions, we selected the following genes: *NRAMP3*, *AT2G32520*, *YSL1*, *GAPC2*, *ZIF1*, *IQD23*, *MT1C*, and *MSD1*. Gene expression analysis was conducted on these genes under both conditions, with error bars representing the standard deviation (n=3). Student's t-test was used to determine the significant differences between the control and cadmium stress conditions, with ** p<0.01 and *** p<0.001.


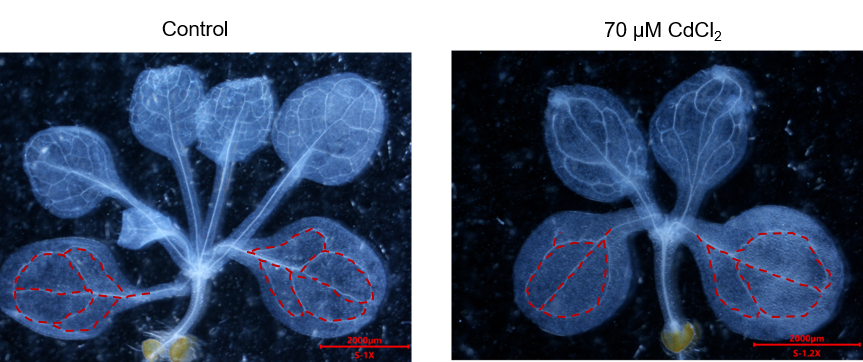


**Fig.S2** Analysis of the developmental status of cotyledon veins in young seedlings under normal and cadmium stress conditions.

After treatment with 1/2 MS and 70 μM CdCl_2_, *Arabidopsis* seedlings were cleaned with chloral hydrate and placed under a phase contrast microscope to observe the development of cotyledon veins. The cotyledon veins were marked with red dashed lines. Scale bar = 2 mm.


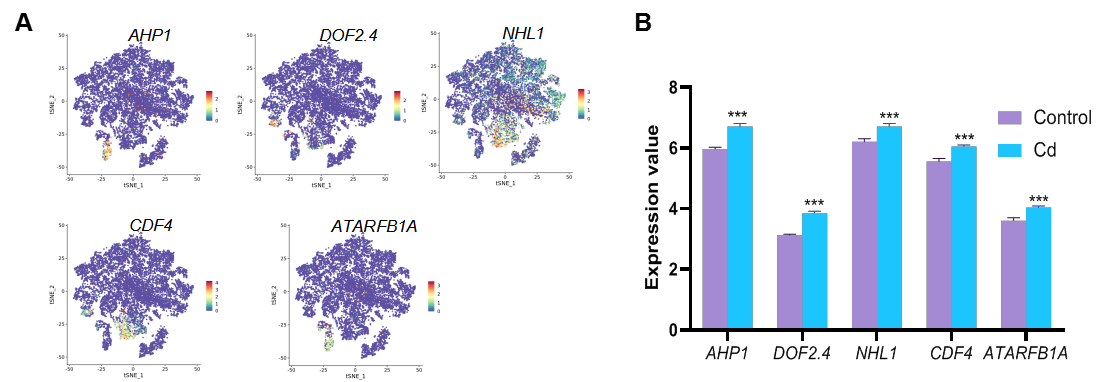


**Fig.S3** Visualization and relative expression analysis of genes specifically expressed in vascular tissues.

1. Visualization of cellular expression of *AHP1*, *DOF2.4*, *NHL1*, *CDF4*, and *ATARFB1A* genes in single-cell data. (B) Relative expression analysis of *AHP1*, *DOF2.4*, *NHL1*, *CDF4*, and *ATARFB1A* gene genes under control and cadmium stress conditions; The error line represents the standard deviation (n=3). The significant difference between control and cadmium stress conditions was determined through Student's t-test (n=3), with *** p<0.001.


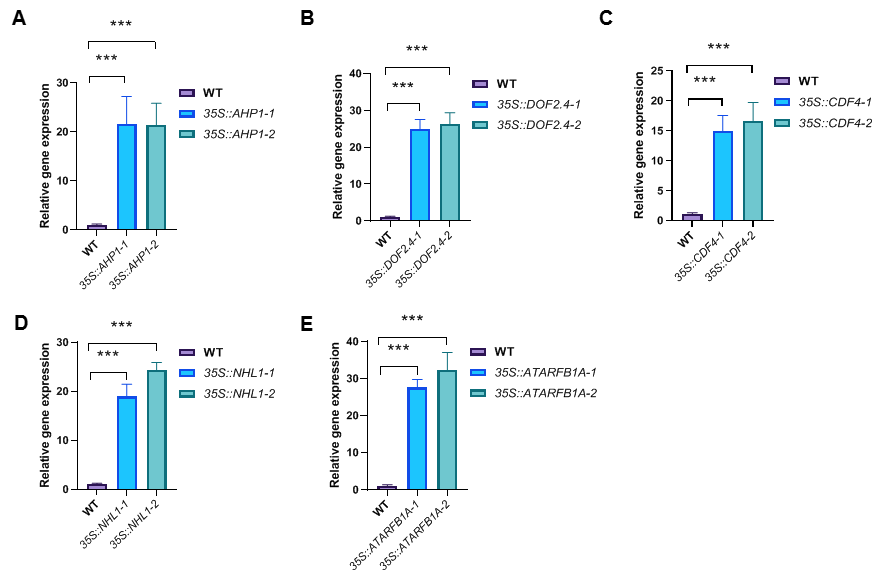


**Fig.S4** Detection of expression levels of *AHP1*, *DOF2.4*, *CDF4*, *NHL1*, and *ATARFB1A* in corresponding transgenic plants. (A) The expression level of *AHP1* in WT, *35S::AHP1-1*, and *35S::AHP1-2* seedlings was analyzed using qPCR. (B) The expression level of *DOF2.4* in WT, *35S::DOF2.4-1*, and *35S::DOF2.4-2* seedlings was analyzed using qPCR. (C) The expression level of *CDF4* in WT, *35S::CDF4-1*, and *35S::CDF4-2* seedlings was analyzed using qPCR. (D) The expression level of *NHL1* in WT, *35S::NHL1-1*, and *35S::NHL1-2* seedlings was analyzed using qPCR. (E) The expression level of *ATARFB1A* in WT, *35S::ATARFB1A-1*, and *35S::ATARFB1A-2* seedlings was analyzed using qPCR. The error line represents the standard deviation (n=3), and the expression levels of *AHP1*, *DOF2.4*, *CDF4*, *NHL1*, and *ATARFB1A* in corresponding transgenic plants were analyzed using Student's t-test to show significant differences compared to the WT. *** p<0.001.


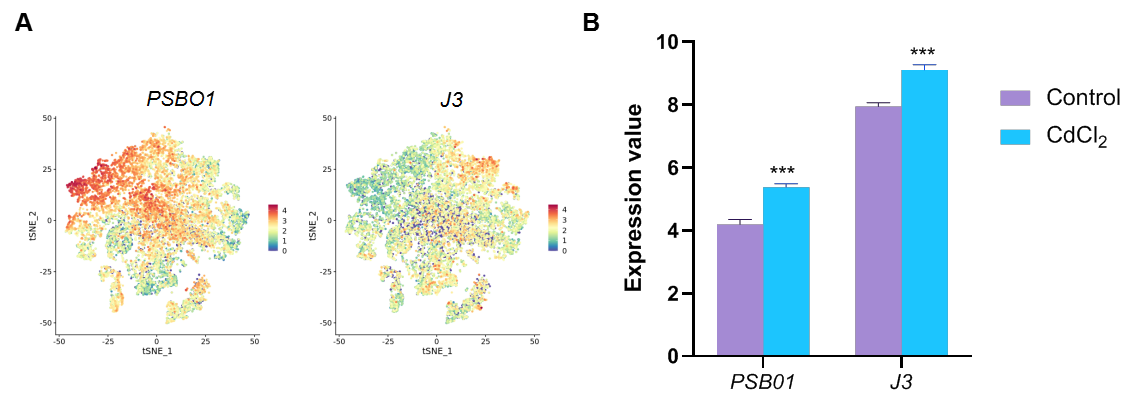


**Fig.S5** Visualization and relative expression analysis of *PSBO1* and *J3* genes in cells.

(A) Visualization analysis of cell expression of *PSBO1* and *J3* in single-cell data. (B) Relative expression analysis of *PSBO1* and *J3* under control and cadmium stress conditions; The error line represents the standard deviation (n=4), and the expression levels of *PSBO1* and *J3* under cadmium stress conditions were analyzed using Student's t-test to show significant differences compared to the control conditions. *** p<0.001.


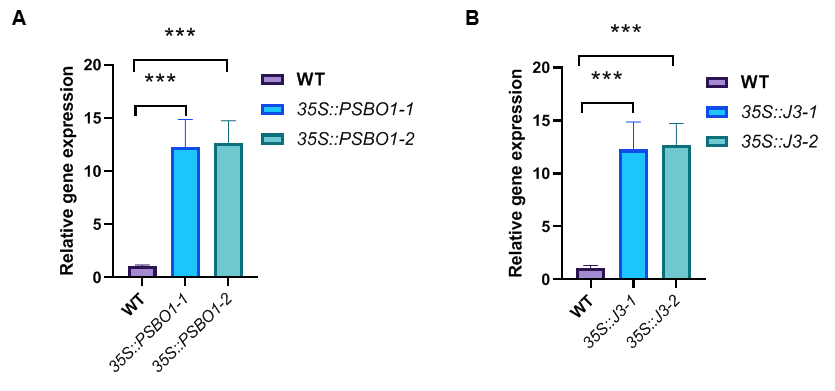


**Fig.S6** Detection of expression levels of *PSBO1* and *J3* in corresponding transgenic plants. (A) The expression level of *PSBO1* in WT, *35S::PSBO1-1*, and *35S::PSBO1-2* seedlings was analyzed using qPCR. (B) The expression level of *J3* in WT, *35S::J3-1*, and *35S::J3-2* seedlings was analyzed using qPCR. The error line represents the standard deviation (n=3), and the expression levels of *PSBO1* and *J3* in corresponding transgenic plants were analyzed using Student's t-test to show significant differences compared to the WT. *** p<0.001.

**
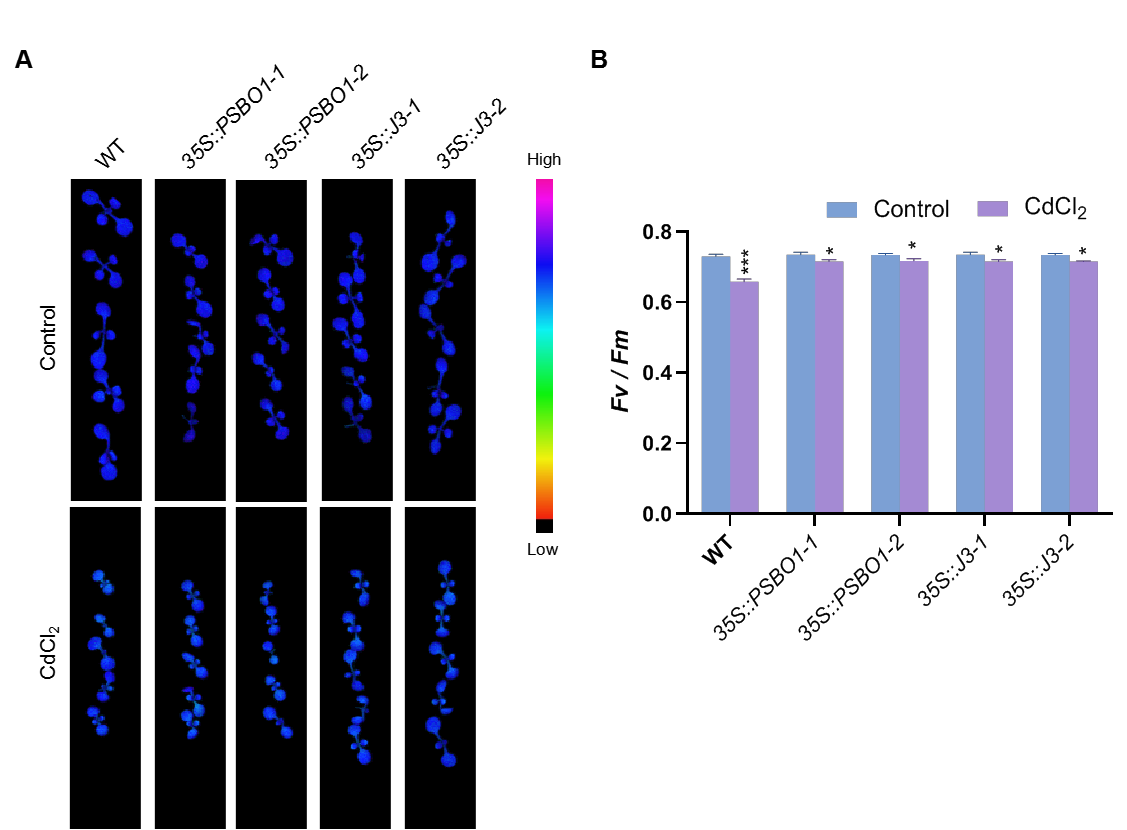
**

**Fig.S7** Chlorophyll fluorescence measurement and statistical analysis of WT, *35S::PSBO1* and *35S::J3* seedlings.

(A) To observe the effect of CdCl_2_ on plant photosynthesis, a chlorophyll fluorescence meter was used to measure the chlorophyll fluorescence of WT, *35S::PSBO1-1*, *35S::PSBO1-2*, *35S::J3-1*, and *35S::J3-2* seedlings grown on control and CdCl_2_ media for 7 days. The images showing their Fv/Fm values are presented in the figure.

(B) Statistical analysis of the Fv/Fm values from (A) was conducted. Error bars represent the standard deviation. Fv/Fm values under normal conditions were used as controls. Student's t-test was performed to analyze the significant differences between cadmium stress conditions and normal conditions (n=5), with *** p<0.001, ** p<0.01, and * p<0.05.


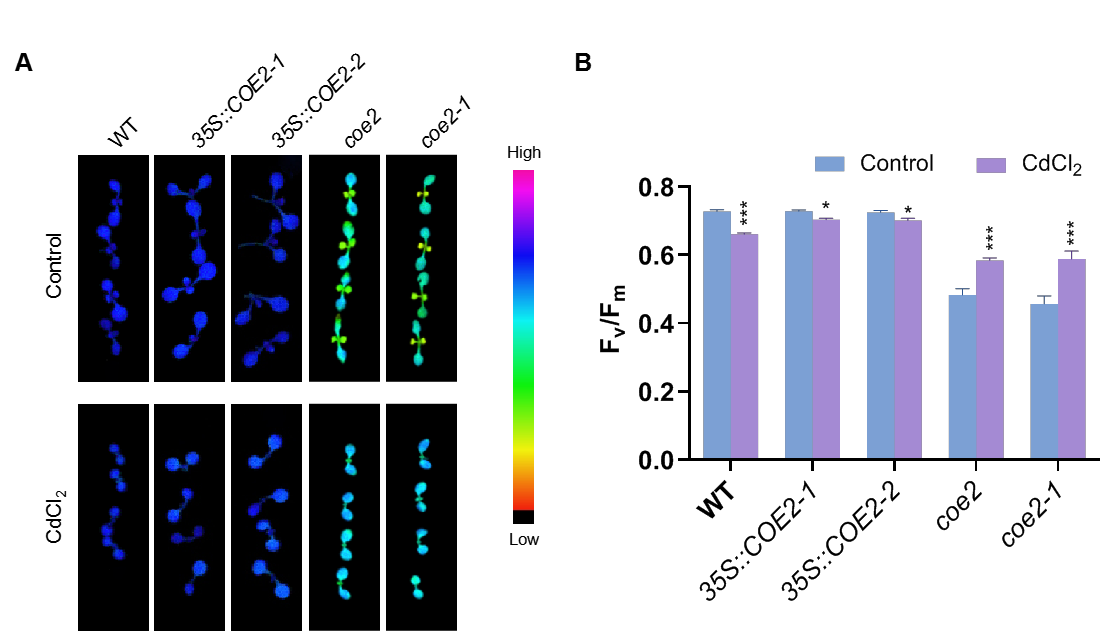


**Fig.S8** Chlorophyll fluorescence measurement and statistical analysis of WT, *35S::COE2-1*, *35S::COE2-2*, *coe2*, and *coe2-1* seedlings.

(A) To observe the effect of CdCl_2_ on plant photosynthesis, a chlorophyll fluorescence meter was used to measure the chlorophyll fluorescence of WT, *35S::COE2-1*, *35S::COE2-2*, *coe2*, and *coe2-1* seedlings grown on control and CdCl_2_ media for 7 days. The images showing their Fv/Fm values are presented in the figure. (B) Statistical analysis of the Fv/Fm values from (A) was conducted. Error bars represent the standard deviation. Fv/Fm values under normal conditions were used as controls. Student's t-test was performed to analyze the significant differences between cadmium stress conditions and normal conditions (n=5), with *** p<0.001, ** p<0.01, and * p<0.05.


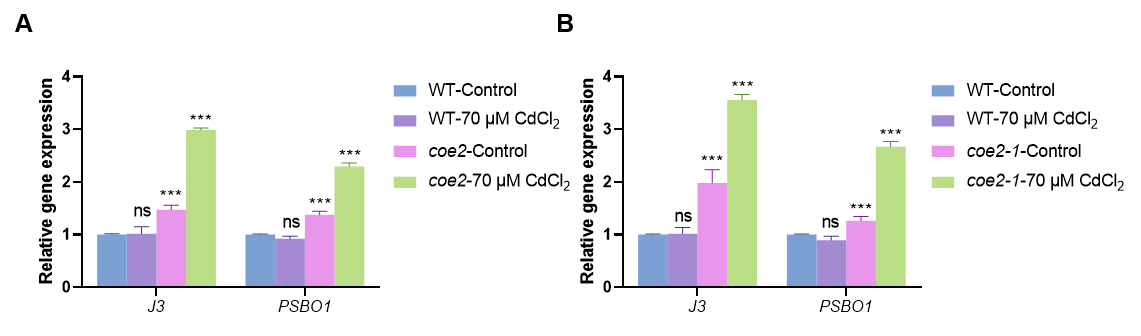


**Fig.S9** Detection of expression levels of *J3* and *PSBO1* in WT, *coe2*, and *coe2-1* seedlings.

The expression levels of *J3* and *PSBO1* in WT, *coe2*, and *coe2-1* seedlings under control and CdCl_2_ treatment conditions were analyzed using qPCR. WT seedlings under control conditions were used as the baseline (value set to 1). The significance of the expression levels of *J3* and *PSBO1* in WT, *coe2*, and *coe2-1* seedlings under different conditions was determined using one-way ANOVA. Error bars represent the standard deviation (n=3). *** p<0.001, and ns indicates no significant difference.


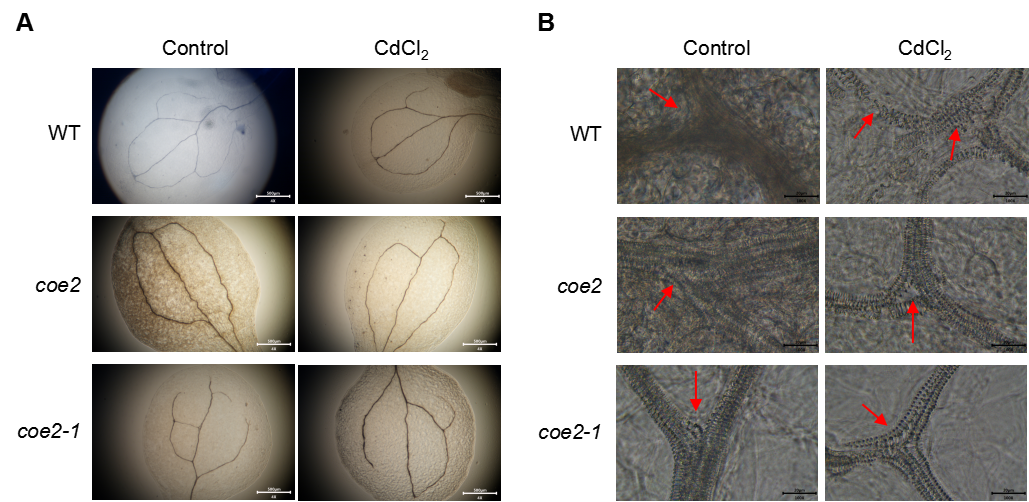


**Fig.S10** Analysis of the effects of CdCl_2_ treatment on the development of leaf vein tissues in WT, *coe2*, and *coe2-1* seedlings.

1. The cotyledons of WT, *coe2*, and *coe2-1* seedlings grown for 7 days under control and CdCl_2_ conditions were cleaned with chloral hydrate, and their vein development was detected using a phase contrast microscope. Scale bar = 500 μm. (B) A detailed view of the leaf vein tissue from (A) was observed, with the red arrow indicating the leaf vein tissue cells. Scale bar = 20 μm.


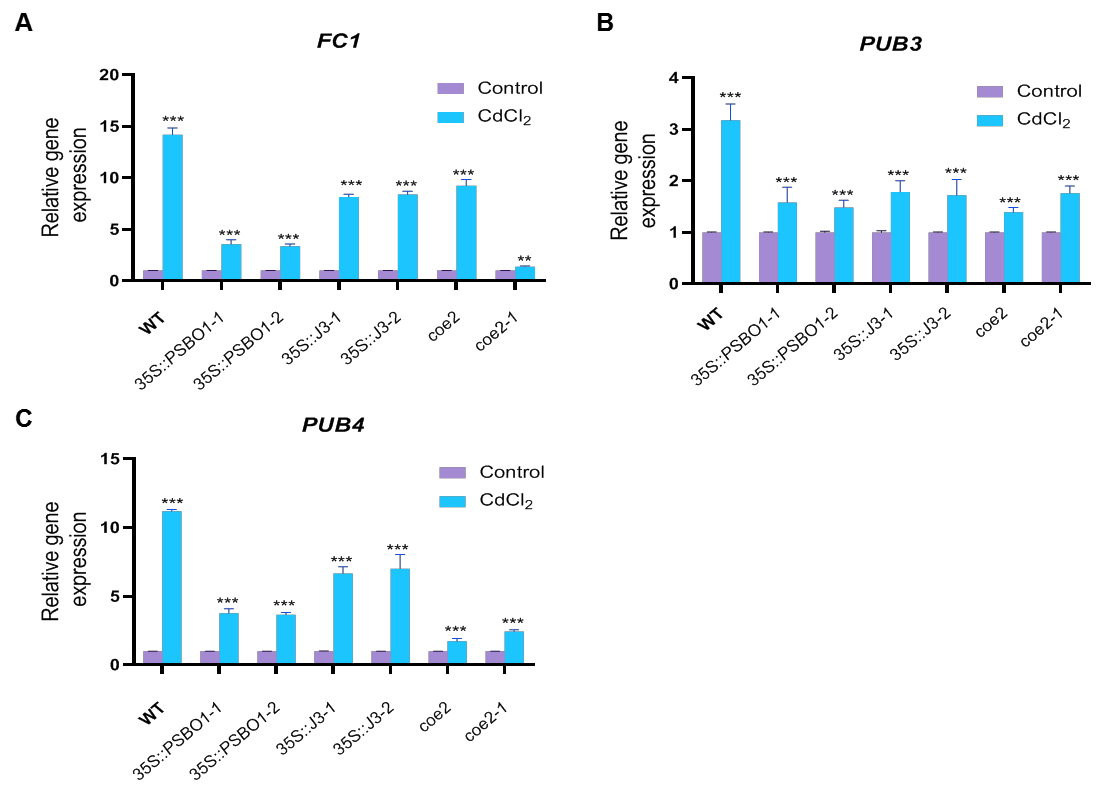


**Fig.S11** Effects of CdCl_2_ treatment on the expression of *FC1*, *PUB3*, and *PUB4*.

(A) The relative expression levels of *FC1* were detected using qPCR in WT, *35S::PSBO1-1*, *35S::PSBO1-2*, *35S::J3-1*, *35S::J3-2*, *coe2*, and *coe2-1* seedlings under both control and CdCl_2_ treatment conditions. (B) The relative expression levels of *PUB3* were measured using qPCR in WT, *35S::PSBO1-1*, *35S::PSBO1-2*, *35S::J3-1*, *35S::J3-2*, *coe2*, and *coe2-1* seedlings under both control and CdCl_2_ treatment conditions. (C) The relative expression levels of *PUB4* were determined using qPCR in WT, *35S::PSBO1-1*, *35S::PSBO1-2*, *35S::J3-1*, *35S::J3-2*, *coe2*, and *coe2-1* seedlings under both control and CdCl_2_ treatment conditions. Significant differences (n=3) between CdCl_2_ treatment and control conditions were determined using Student's t-test, *** p<0.001.
